# Membrane phospholipid alteration causes chronic ER stress through early degradation of homeostatic ER-resident proteins

**DOI:** 10.1038/s41598-019-45020-6

**Published:** 2019-06-14

**Authors:** Peter Shyu, Benjamin S. H. Ng, Nurulain Ho, Ruijie Chaw, Yi Ling Seah, Charlie Marvalim, Guillaume Thibault

**Affiliations:** School of Biological Sciences, Nanyang Technological University, Singapore, 637551 Singapore

**Keywords:** Phospholipids, Endoplasmic reticulum

## Abstract

Phospholipid homeostasis in biological membranes is essential to maintain functions of organelles such as the endoplasmic reticulum. Phospholipid perturbation has been associated to cellular stress responses. However, in most cases, the implication of membrane lipid changes to homeostatic cellular response has not been clearly defined. Previously, we reported that *Saccharomyces cerevisiae* adapts to lipid bilayer stress by upregulating several protein quality control pathways such as the endoplasmic reticulum-associated degradation (ERAD) pathway and the unfolded protein response (UPR). Surprisingly, we observed certain ER-resident transmembrane proteins, which form part of the UPR programme, to be destabilised under lipid bilayer stress. Among these, the protein translocon subunit Sbh1 was prematurely degraded by membrane stiffening at the ER. Moreover, our findings suggest that the Doa10 complex recognises free Sbh1 that becomes increasingly accessible during lipid bilayer stress, perhaps due to the change in ER membrane properties. Premature removal of key ER-resident transmembrane proteins might be an underlying cause of chronic ER stress as a result of lipid bilayer stress.

## Introduction

Phospholipid homeostasis is crucial in the maintenance of various cellular processes and functions. Phospholipids participate extensively in the formation of biological membranes, which give rise to distinct intracellular compartments known as organelles for metabolic reactions, storage of biomolecules, signalling, as well as sequestration of metabolites. Existing as various and distinct species, phospholipids are regulated within relatively narrow limits and their composition in biological membranes among organelles differs significantly^1^.

Perturbation of two of the most abundant phospholipids, phosphatidylcholine (PC) and phosphatidylethanolamine (PE), correlate to disease states including non-alcoholic fatty liver disease (NAFLD)^2–5^, type II diabetes (T2D)^6^, as well as cardiac and muscular dystrophies^7^. As PC and PE form the bulk of biological membranes, the perturbation of PC and PE levels ultimately results in lipid bilayer stress, which in turn causes endoplasmic reticulum (ER) stress^8,9^. For instance, an elevated PC/PE ratio in obesity was found to contribute to the development of NAFLD^10,11^. Phospholipid perturbation was shown to cause the premature degradation of the sarco/endoplasmic reticulum Ca^2+^-ATPase (SERCA) ion pump, therefore disrupting calcium homeostasis and resulting in chronic ER stress^10^. This eventually led to hepatic steatosis and liver failure. In another study, mice fed with high fat diet exhibited an increase in gut microbiota enzymatic activity that has been shown to reduce choline^12,13^, an essential dietary nutrient primarily metabolised in the liver and used for the synthesis of PC. Therefore, better understanding the events leading toward the development of chronic ER stress upon phospholipid bilayer stress (hereafter referred to as lipid bilayer stress; LBS) is relevant to physiological conditions.

**Figure 1:**
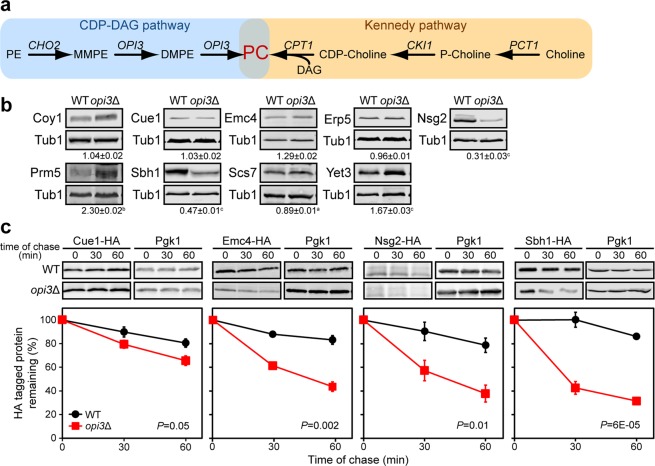
A subset of ER transmembrane proteins is prematurely degraded under lipid imbalance. (**a**) Metabolic pathway for the synthesis of phosphatidylcholine in *S. cerevisiae*. PE, phosphatidylethanolamine; MMPE, *N*-monomethyl phosphatidylethanolamine; DMPE, *N*,*N*-dimethyl phosphatidylethanolamine; PC, phosphatidylcholine; DAG, diacylglycerol; CDP-choline, cytidine diphosphate-choline; P-choline, phosphate-choline. **(b)** Steady state level of transmembrane proteins. Equal cell numbers were harvested. Proteins were separated by SDS-PAGE and detected by immunoblotting with antibodies against the HA tag and Tub1 as loading control. ^a^*P* < 0.05, ^b^*P* < 0.01, ^c^*P* < 0.005. **(c)** Degradation of HA-tagged proteins was analysed after blocking protein translation with cycloheximide. Proteins were separated by SDS-PAGE and detected by immunoblotting with antibodies against the HA tag and Pgk1 as loading control. Data shown is the mean ± SEM (n = 3). All uncropped immunoblot images are included in the Supplementary File. Statistical analyses were subjected to paired two-tailed Student’s t-test.

In *Saccharomyces cerevisiae*, *de novo* synthesis of PC is catalysed by the enzymes Cho2 and Opi3, and this process is similarly carried out by the Opi3 homologue, PEMT, in mammals (Fig. 1a). Cho2 first methylates PE to *N*-monomethyl phosphatidylethanolamine (MMPE), which is further methylated by Opi3 to PC through the intermediate *N*,*N*-dimethyl phosphatidylethanolamine (DMPE). Alternatively, PC is synthesised from choline, when available, through the Kennedy pathway. Both the *de novo* and Kennedy pathways are highly conserved from yeast to humans. In the absence of PEMT, dietary choline is essential to prevent NAFLD^5^. Previously, we developed a lipid bilayer stress yeast model to recapitulate a major contributor to NAFLD pathophysiology by deleting the gene *OPI3*^14^.

The unfolded protein response (UPR) is a stress response pathway monitoring ER stress to restore cellular homeostasis^15^. Upon accumulation of misfolded proteins within the ER lumen, the UPR is activated and alleviates stress by reversing severe dysfunctions through the upregulation of nearly 400 target genes in yeast cells^16^. Major targeted regulatory pathways includes cytosolic protein quality control (CytoQC), ER-associated degradation (ERAD), protein translocation, post-translational modification and phospholipid biosynthesis^16,17^. Through a general attenuation of protein translation, together with the enhanced clearance of misfolded proteins and protein folding capacity^18^, the UPR aims to achieve ER homeostasis.

In addition to proteotoxic stress, the UPR is essential in alleviating ER stress in lipid dysregulated cells to maintain protein biogenesis, protein quality control and membrane integrity^14,19–21^. Recently, it was demonstrated that the N-terminal transmembrane domain of the sole yeast ER stress sensor Ire1 functions as an amphipathic helix to detect biophysical changes at the ER membrane^22^. Lipid bilayer stress, by the deletion of *CHO2* or *OPI3*, is synthetically lethal with *IRE1* as well as its downstream transcription factor *HAC1*^20,21^. Lipid bilayer stress has been well characterised to induce ER stress^23–25^, and the failure of the UPR to restore lipid homeostasis might be implicated in human diseases^26–28^. This clearly establishes the critical role of the UPR in buffering the lethal effects of lipid bilayer stress to ensure cell survival.

In this study, we observed certain ER-resident transmembrane proteins, part of the UPR programme, to be prematurely degraded specifically under lipid bilayer stress. First, we demonstrated that lipid bilayer stress affects the ER membrane, which results in the destabilisation of transmembrane proteins. Furthermore, we elucidated the mechanism of how one such transmembrane protein, Sbh1, is recognised for degradation through ERAD. Our findings indicate that under lipid bilayer stress, the degron of free Sbh1 becomes more readily accessible to the Doa10 complex, thus leading to its premature degradation.

## Results

### A subset of transmembrane proteins is destabilised during lipid bilayer stress

Global transcriptional and proteomic analyses from our previous work indicated a dramatically altered biochemical landscape in yeast cells under lipid bilayer stress^14^. Among these, 66 proteins were identified to be transcriptionally upregulated yet displayed a decrease in protein abundance (Supplementary Table S1), including 11 ER-resident transmembrane proteins. From these, we analysed the steady-state levels of ten transmembrane protein candidates in cells under lipid bilayer stress using the PC-deficient *opi3*∆ strain^14^ (Fig. 1a,b). Coy1, Cue1 and Erp5 exhibited similar protein steady-states in *opi3*∆ and WT, while Nsg2, Sbh1 and Scs7 had significantly lower steady-state levels in *opi3*∆. Surprisingly, Emc4, Prm5 and Yet3 showed higher steady-state protein levels. To exclude possible cellular functions that could be grossly affected from lipid bilayer stress such as transport and secretion, we focused on the ER-resident proteins Cue1, Emc4, Nsg2, and Sbh1. Cue1 is an essential component of the ERAD pathway^29^. Emc4 is a member of the conserved ER transmembrane complex (EMC) and is required for efficient folding of proteins in the ER^23,30^, including the insertion of tail-anchored ER membrane proteins^31^. The EMC is also proposed to facilitate the transfer of phosphatidylserine from the ER to mitochondria^32^. Nsg2 regulates the sterol-sensing protein Hmg2^33^. Lastly, the β subunit of the Sec61 ER translocon complex, Sbh1, is highly conserved in eukaryotes and plays a role in the translocation of proteins into the ER^34–36^. While Sbh1 is non-essential for translocation, its absence leads to a defect in this process when deleted in conjunction with its paralogue, Sbh2^37^.

To assess the stability of these four transmembrane protein candidates during lipid bilayer stress, a cycloheximide chase assay was performed in WT and *opi3*Δ strains. Half-lives of Emc4, Nsg2, and Sbh1 were found to be significantly reduced under lipid bilayer stress (Fig. 1c). No significant decrease in Cue1-HA protein levels was detected in *opi3*Δ although the decrease was reproducible. One hour after attenuating protein translation, levels of Emc4, Nsg2, and Sbh1 were found to be 27%, 41%, and 58% lower in *opi3*Δ, respectively, compared to WT. This suggests that the UPR programme transcriptionally upregulates genes to restore ER homeostasis under lipid bilayer stress, while the corresponding encoded transmembrane proteins are rapidly recognised and targeted for degradation.

### A subset of ER-localised transmembrane proteins is destabilised by a decrease in phosphatidylcholine

**Figure 2:**
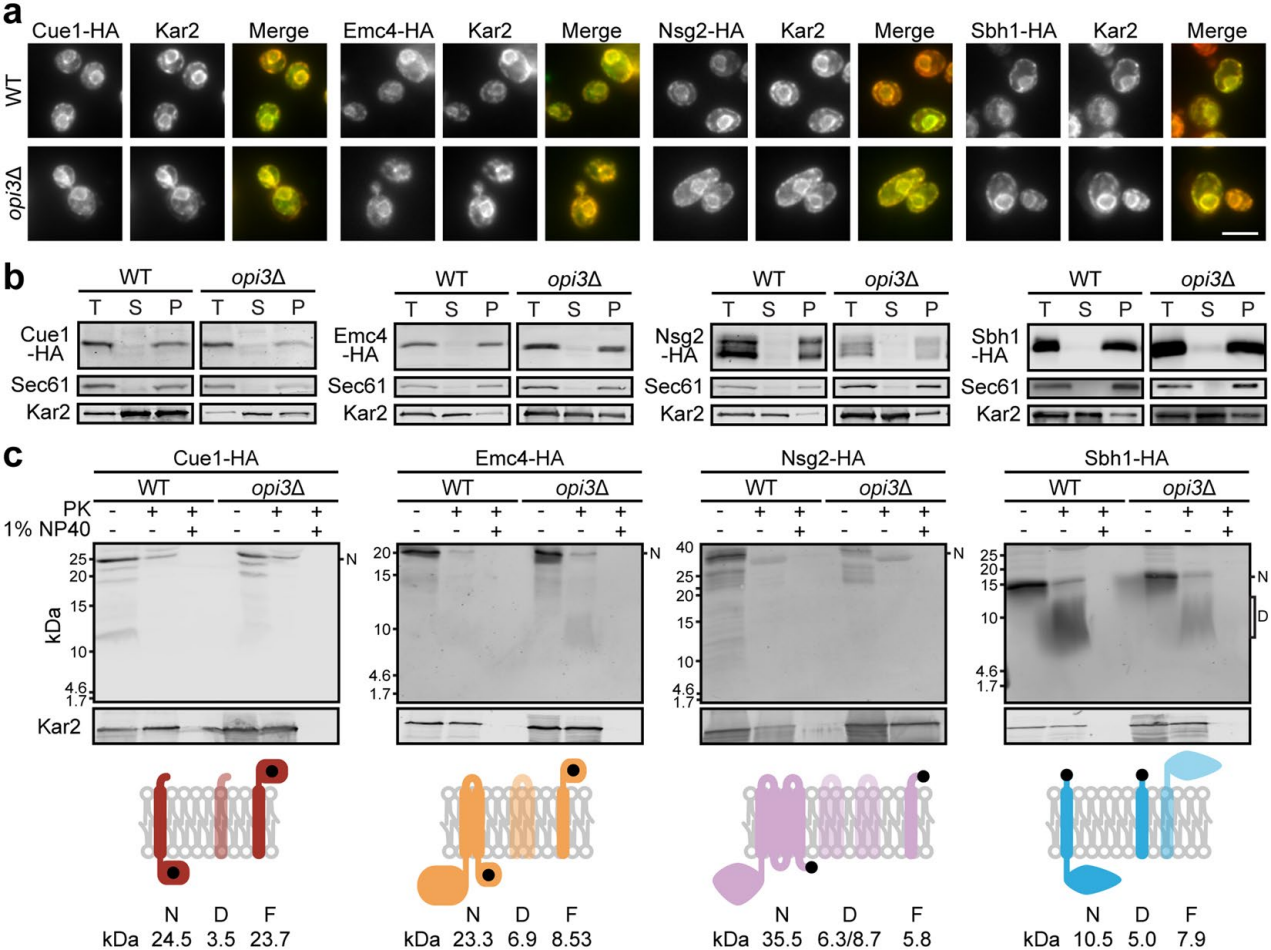
Transmembrane proteins are destabilised by the decrease in phosphatidylcholine synthesis. (**a**) Protein candidates were detected using antibodies against the HA tag and Kar2 as the ER marker. Scale bar, 5 µm (n = 3). **(b)** Membranes prepared from wild-type and *opi3*Δ cells expressing HA-tagged proteins were treated with 0.1 M sodium carbonate, pH 11, for 30 min on ice. A portion was kept as the total fraction (T), and the remaining was subjected to centrifugation at 100,000 × *g*. Supernatant (S) and membrane pellet (P) fractions were collected and analysed by immunoblotting. Proteins were detected using antibodies against HA. Kar2 and Sec61 serve as soluble and integral membrane protein controls, respectively. Images shown are representatives of three independent experiments. **(c)** Membranes prepared from WT and *opi3*Δ cells expressing HA-tagged proteins were treated with 1 mg/ml proteinase K, for 30 min at 37 °C, with or without 1% NP40. HA-tagged proteins were precipitated with 10% TCA, separated by SDS-PAGE and detected by immunoblotting with antibodies against HA. Expected protein molecular weights are shown below for non-digested (N), digested (D), and flipped and digested (F). The orientation of the HA tag is represented as a black dot. Fragments missing the HA tag are therefore undetectable are illustrated with decreased opacity. The ER lumen and cytosol are at the top and bottom of the membrane, respectively. Images shown are representatives of three independent experiments. All uncropped immunoblot images are included in the Supplementary File.

To ensure that Cue1, Emc4, Nsg2, and Sbh1 remain as integral ER membrane proteins during lipid bilayer stress, we verified their localisation at the ER (Fig. 2a) and their insertion into cellular membranes (Fig. 2b) in *opi3*Δ cells. Together, these results suggest that integration into the ER membrane is unaffected by PC depletion. To study the topology of these four proteins, we performed proteinase K (PK) digestion from isolated microsomes (Fig. 2c). In WT cells, the C-terminal HA-tag of Cue1-HA, Emc4-HA and Nsg2-HA are oriented towards the cytosol. Thus, the HA epitope is cleaved off from proteins with a proper topology, while the detection of a HA epitope-bearing peptide after PK digestion indicates an inverted topology. The three proteins were found to be fully digested under lipid bilayer stress and the predicted smaller protein fragments of 23.7, 8.53, and 5.8 kDa were not detected for Cue1-HA, Emc4-HA, and Nsg2-HA, respectively, in both WT and *opi3*Δ. Sbh1-HA is a tail-anchored protein where the C-terminal HA tag is found in the ER lumen. The predicted protein fragment of 10.5 kDa, after PK, digestion was detected in both WT and *opi3*Δ strains, indicative of its correct membrane topology. Typically, tail-anchored proteins are tagged at the N-terminal as the C-terminal interacts with the GET complex for insertion into the ER membrane^38^. This result, along with alkaline carbonate extraction (Fig. 2b), show that adding a C-terminal HA tag to Sbh1 does not interfere with its integration into the ER membrane. The four transmembrane proteins were fully digested in the presence of the non-ionic detergent Nonidet P-40 (NP40). Together, these findings suggest that the four transmembrane proteins are prematurely targeted for degradation after they are fully translated and integrated into the ER membrane during lipid bilayer stress.

**Figure 3:**
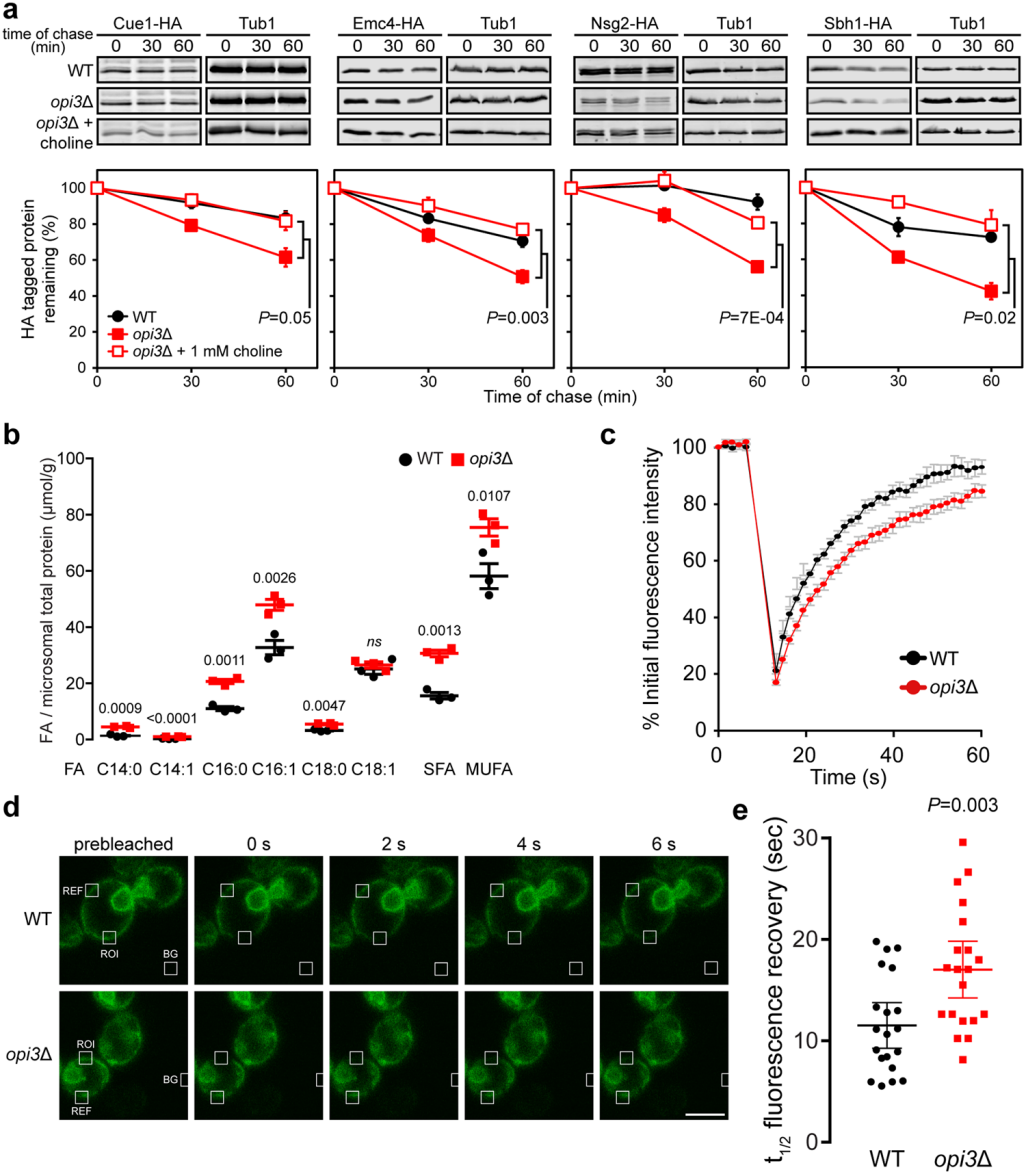
Sbh1 is destabilised from increased membrane fluidity of the ER membrane. (**a**) Cells were grown with or without 1 mM choline before addition of cycloheximide. Time points were taken as indicated. Proteins were separated by SDS-PAGE and detected by immunoblotting with antibodies against the HA tag and Tub1 as loading control (n = 3). **(b)** The abundance of different fatty acids (FA) species in WT and *opi3*Δ microsomal extracts were quantified by gas chromatography with FAME derivatisation. FAs are categorised based on chain length and degree of saturation. SFA, saturated fatty acid; MUFA, monounsaturated fatty acid. Data shown is the mean ± SEM (n = 3). Statistical analysis was subjected to paired two-tailed Student’s t-test. *ns*, non-significant. (**c**–**e**) Fluorescence recovery after photobleaching using Sec63-sGFP in WT and *opi3*Δ cells. (**c**) Averages of Sec63-sGFP signal intensities from 20 cells are plotted over a 60-second period. (**d**) Fluorescence intensity was monitored from areas marked with white boxes for ROI (regions of interest), REF (reference), and BG (background). Scale bar, 5 µm. A region of the cortical ER of live cells was photobleached and recovery points at 1.57 s intervals were taken. (**e**) The time elapsed for the half-maximal fluorescence recovery (t_½_) was calculated and plotted (n = 20). Data shown is the mean ± SEM. All uncropped immunoblot images are included in the Supplementary File. Statistical analysis was subjected to unpaired two-tailed Student’s t-test.

To further establish that the four transmembrane proteins are destabilised specifically from low PC levels, their degradation was monitored in *opi3*Δ cells grown in the presence of choline to restore PC homeostasis^14,39^ (Fig. 1a). Choline supplementation significantly stabilised Cue1-HA, Emc4-HA, Nsg2-HA, and Sbh1-HA in *opi3*Δ to WT levels (Fig. 3a). Subsequently, we concentrated our effort on Sbh1 as a model substrate to better understand how these transmembrane proteins are targeted for premature degradation during lipid bilayer stress.

The UPR is strongly activated in response to lipid bilayer stress^14,23^. In *opi3*Δ cells, UPR activation is constitutively elevated and unresolved, thereby giving rise to chronic ER stress^14,40^. To ensure that Sbh1 is not destabilised as a consequence of strong UPR activation, we expressed a constitutively active form of the downstream effector, *HAC1*^*i*^, into WT cells^17,41^. As expected, *HAC1*^*i*^-induced UPR did not lead to the destabilisation of Sbh1 in WT cells (Supplementary Fig. S1a). Noticeably, the steady-state level of Sbh1 is higher in UPR-activated WT cells as *SBH1* is upregulated by the UPR programme^14,16^. Additionally, yeast cells can mount an intact UPR in the absence of *SBH1* (Supplementary Fig. S1b). These collectively indicate that the elevated UPR activation in *opi3*Δ cells alone is not sufficient to drive premature Sbh1 degradation, and that other gross changes within the cellular landscape may cause this phenomenon.

### Changes in ER membrane fluidity is sufficient to destabilise Sbh1

To narrow down the specific effect of lipid bilayer stress that could contribute to the premature degradation of Sbh1, we analysed the fatty acid (FA) composition of whole cells and fractionated microsomes. Our analyses indicated a significant increase in FA abundance in the microsomes of *opi3*Δ cells compared to those of WT (Fig. 3b), likely resulting from the UPR-induced ER expansion associated with this mutant^14^. A similar increase in FAs was observed in *opi3*Δ at the cellular level (Supplementary Fig. S2a), potentially due to the build-up of triacylglycerol stored in cytosolic lipid droplets upon UPR activation^14,42,43^. Furthermore, we found no marked change in the saturation state of FAs in *opi3*Δ cells compared to WT (Fig. 3b).

We have previously reported that the *opi3*Δ mutant does not orchestrate a compensatory response to lipid changes but is instead characterised by an altered protein homeostatic network. However, with the inability of *opi3*Δ cells to synthesise PC, the accumulation of structurally varied intermediates may lead to changes in the biophysical properties of the membrane. In support of this, a substantial increase in MMPE (Fig. 1a) is expected to induce negative membrane curvature stress as what has been reported for PE^44^.

To better understand the impact of membrane phospholipid remodelling on the behaviour of transmembrane proteins, we monitored the dynamics of the ER-resident membrane protein Sec63-sfGFP by fluorescence recovery after photobleaching (FRAP)^45^. A region of the cortical ER is photobleached and signal recovery correlates with Sec63-sfGFP mobility. The recovery of Sec63-sfGFP fluorescence was significantly slower in *opi3*Δ compared to WT suggesting rigidity of the ER membrane (Fig. 3c–e). This result is consistent with previous reports on the effect of decreased PC/PE ratio in membrane stiffening^46,47^. Sec63-sfGFP stability was not affected by lipid bilayer stress as predicted from our previous proteomic analysis^14^ (Supplementary Fig. S2b). Taken together, these suggest that a decrease in membrane fluidity could prevent transmembrane proteins from associating with their interacting partners following translation and further result in premature degradation.

### Sbh1 binding to interacting partners is compromised under lipid imbalance

**Figure 4:**
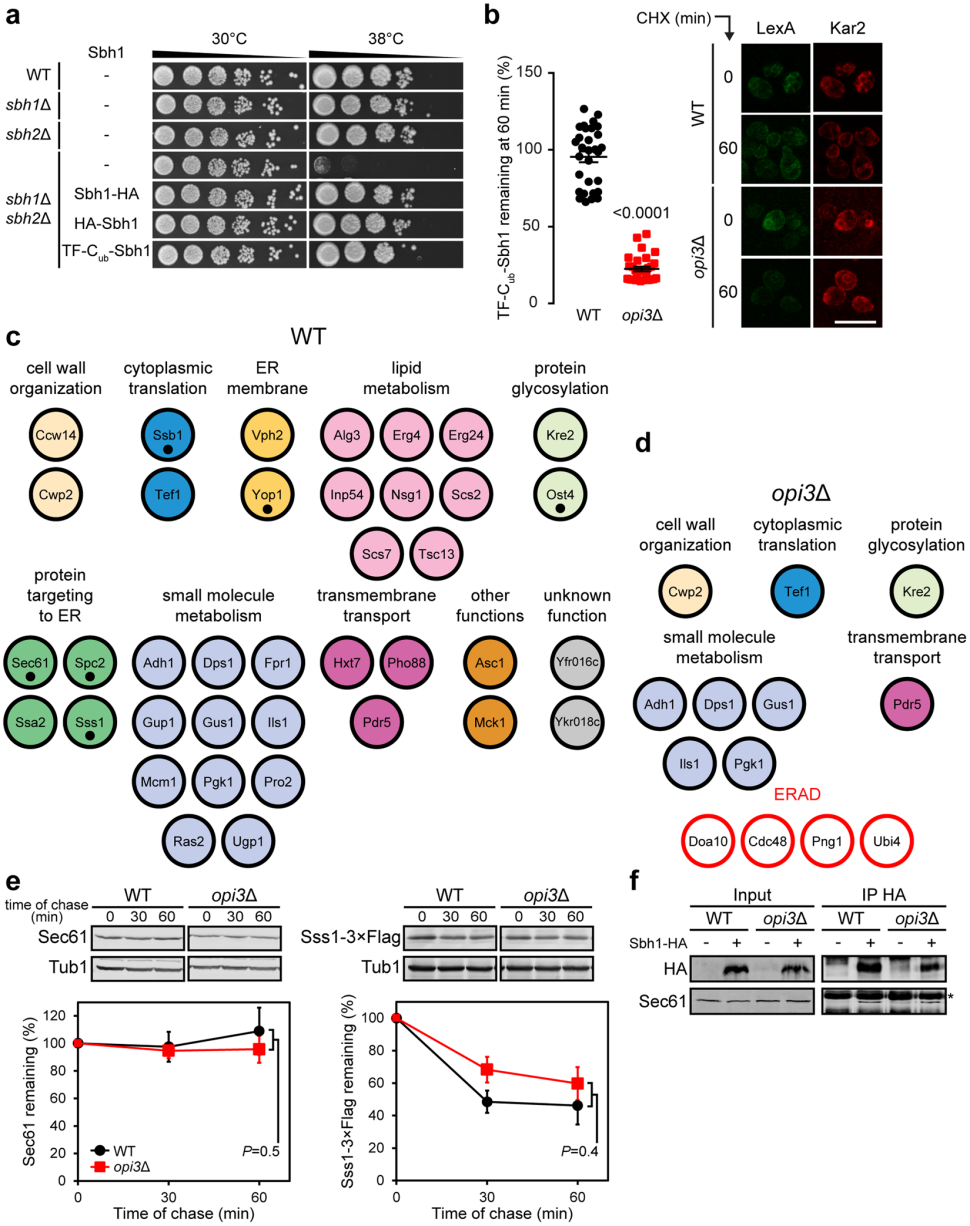
Sbh1 binding to interacting partners is compromised under lipid imbalance. (**a**) Strains harbouring vector controls or plasmids encoding epitope tagged Sbh1 were grown to saturation at 30 °C, and serial dilutions were prepared and further spotted onto SC-LEU plates. Strains were incubated at the indicated temperatures until the appearance of colonies. **(b)** Degradation of TF-C_ub_-Sbh1 was analysed in WT and *opi3*Δ cells 60 min after treatment with cycloheximide through immunofluorescence. Fluorescence intensities were normalised against Kar2 and are presented as percentages relative to the zero-time point. Representative images for three biological replicates are shown. Scale bar, 10 µm. Data shown is the mean ± SEM (n = 30). Statistical analysis was subjected to unpaired two-tailed Student’s t-test. (**c,d**) Proteins identified as interacting partners of TF-C_ub_-Sbh1 through the MYTH screen in WT (**c**) and *opi3*Δ (**d**) cells. ERAD factors were only detected in *opi3*Δ and are denoted in red. Previously reported interactors of Sbh1 are indicated with black dots. **(e)** The degradation of Sec61 or Sss1-3 × Flag was analysed in WT and *opi3*Δ cells after blocking translation with cycloheximide. Proteins were separated by SDS-PAGE and detected by immunoblotting with antibodies against Sec61 or the Flag tag and Tub1 as loading control. **(f)** Immunoprecipitation of Sbh1-HA with protein G beads were analysed in WT and *opi3*Δ native cell lysates. Eluted and input fractions were resolved by SDS-PAGE, transferred to nitrocellulose membrane, and analysed by immunoblotting with antibodies against Sec61 and the HA tag. All uncropped immunoblot images are included in the Supplementary File.

To further characterise the effect of lipid bilayer stress on Sbh1 stability, we performed the split-ubiquitin based membrane yeast two hybrid (MYTH) screen in WT and *opi3*Δ cells to identify changes in Sbh1 membrane protein interactome^48,49^. As opposed to our C-terminally tagged Sbh1-HA, the reporter moiety must be fused to the N-terminal cytosolic domain of Sbh1 (TF-C_ub_-Sbh1) for compatibility with the MYTH assay. Thus, we assessed the functionality of the N-terminal MYTH reporter-tagged Sbh1 *vis-à-vis* Sbh1 constructs tagged in either the N- or C-terminal in *sbh1*Δ*sbh2*Δ mutant cells at 38 °C (Fig. 4a). The temperature-sensitive growth defect of the *sbh1*Δ*sbh2*Δ mutant at 38 °C can be rescued with either a functional Sbh1 or Sbh2 as previously reported^50^. Sbh1-HA, HA-Sbh1, and TF-C_ub_-Sbh1 were all sufficient to rescue the growth defect of the *sbh1*Δ*sbh2*Δ mutant, suggesting that Sbh1 is functional regardless of epitope tag position. Despite the presence of a small HA tag at the C-terminal, the tail-anchored protein Sbh1 remains inserted into the ER membrane through the GET complex^51^ and interacts with the Sec61 complex^52,53^ (Supplementary Fig. S3a). Surprisingly, we found that the N-terminally tagged HA-Sbh1 protein was highly stable under lipid bilayer stress (Supplementary Fig. S3b), thereby raising the possibility that TF-C_ub_-Sbh1 may be equally stable in *opi3*Δ cells as they are in WT. We then proceeded to validate the stability of TF-C_ub_-Sbh1 under lipid bilayer stress through immunofluorescence signal quantification following cycloheximide treatment. One hour after attenuation of protein synthesis, the TF-C_ub_-Sbh1 bait protein was destabilised in *opi3*Δ cells to 24% of WT (Fig. 4b) similar to results obtained with Sbh1-HA (Fig. 3b). These findings support the suitability of TF-C_ub_-Sbh1 to investigate potential changes in protein interactors that could lead to the premature degradation of Sbh1 as a model transmembrane substrate under lipid bilayer stress.

To carry out the MYTH assay, strains expressing the Sbh1 bait protein were transformed with a yeast prey genomic plasmid library in which open reading frames are fused to sequences encoding the cognate reporter moiety^54^. A total of 49 and 14 putative Sbh1-interacting proteins were identified in WT and *opi3*Δ, respectively (Supplementary Fig. S3c). To eliminate false positive interactors, a bait dependency test was done against the unrelated single-pass transmembrane domain of the human T-cell surface glycoprotein CD4 fused to C_ub_-LexA-VP16^54^. In WT, we identified 38 bona fide Sbh1 interactors, including those that have previously been reported such as Ost4, Sec61, Spc2, Ssb1, Sss1, and Yop1^52,55–57^ (Fig. 4c). Sbh1 was also found to interact with membrane proteins involved in sterol biogenesis (Erg4, Erg24 and Nsg1) and fatty acid elongation (Elo2 and Tsc13). On the other hand, only 13 proteins were found to interact with Sbh1 in *opi3*Δ cells (Fig. 4d). No interaction of Sbh1 with Sec61 and Sss1 was detected in *opi3*Δ. This suggests that Sbh1 could be dissociated from the Sec61 complex under lipid bilayer stress which leads to its premature degradation. This is consistent with the finding that Sbh2, the paralogue of Sbh1, becomes destabilised and rapidly degraded when unbound to the Sec61-like complex Ssh1^50^. Similarly, Sbh1 was found to interact with proteins of the ERAD pathway under lipid bilayer stress (Fig. 4d). These include the membrane-embedded ubiquitin-protein ligase Doa10 which is part of the ERAD Doa10 complex^58,59^. As the Doa10 complex is generally specific for substrates containing cytosolic lesions (ERAD-C)^60^ or intramembrane degrons^61^, it suggests that a polypeptide stretch of Sbh1 might become exposed on its cytosolic side under lipid bilayer stress, thus making it susceptible to ubiquitination. Alternatively, the Sbh1 α-helix may become more accessible for recognition by the Doa10 complex in *opi3*Δ cells. Subsequently, targeted substrates for degradation are polyubiquitylated in the cytosol by the addition of ubiquitin (Ubi4)^62^, a protein identified to interact with Sbh1 exclusively in *opi3*Δ cells. The AAA^+^ ATPase protein Cdc48 was also found to interact with Sbh1 in *opi3*Δ cells (Fig. 4d). Ubiquitylated substrates are retro-translocated to the cytosol by the action of the Cdc48 complex and targeted to the proteasome for degradation^63,64^. Another important player of the ERAD pathway, Png1, was found to exclusively interact with Sbh1 under lipid bilayer stress. Png1 catalyses the deglycosylation of misfolded glycoproteins, which is a critical step for ERAD substrate modification for subsequent proteasomal degradation^65^. Furthermore, Png1 has been found to interact directly with ERAD and proteasomal components in both yeast and mammalian cell systems^66,67^. While yeast Png1 was found to be non-essential for the turnover of the misfolded glycoprotein model substrate carboxypeptidase Y (CPY*), its gene deletion significantly increased the half-life of CPY* in a deglycosylation-independent manner^65^. Therefore, it could not be excluded that Sbh1 as a non-glycosylated protein is destabilised under lipid bilayer stress partly through an increased interaction with Png1. Together, the MYTH interactor screen results suggest that a change in membrane properties lead to the dissociation of Sbh1 from the Sec61 complex, resulting in its rapid degradation through the ERAD-C complex.

To ensure levels of the Sec61 complex subunits, apart from Sbh1, remain unchanged under lipid bilayer stress, we carried out the cycloheximide chase assay to follow the stability of Sec61 and Sss1-3 × Flag. Both Sec61 and Sss1 were found to be as stable in *opi3*Δ as they are in WT, in agreement with our previous proteomic data^14^ (Fig. 4e). To assess the interaction of Sbh1 with Sec61 complex on the ER membrane under lipid bilayer stress, native co-immunoprecipitation (co-IP) was performed (Fig. 4f). In contradiction to the MYTH screen results, Sec61 was found to interact stably with Sbh1-HA in both WT and *opi3*Δ strains. The discrepancy could be due to the difference in membrane dynamics *in vivo* and *in vitro* from the MYTH and co-IP assay, respectively. Interestingly, Sbh1-HA was significantly stabilised in *opi3*Δ cells devoid of endogenous Sbh1 (*sbh1*Δ*opi3*Δ) (Supplementary Fig. S3d). While Sbh1-HA remains functional (Fig. 4a), the presence of a terminal epitope tag may reduce its affinity with cognate protein interactors such as Sec61, as it has been reported for other terminal-epitope tag fusion proteins^68,69^. However, in the absence of its endogenous counterpart, Sbh1-HA could increasingly interact with Sec61, indicative of a binding-dependent stabilisation reminiscent of Sbh2^50^. Taken together, these results suggest that the loss of interaction between Sbh1 and the Sec61 complex prompts its recognition by the Doa10 complex for destruction by ERAD.

### Sbh1 is atypically destabilised through Doa10 independently from its cytosolic lysine residues

**Figure 5:**
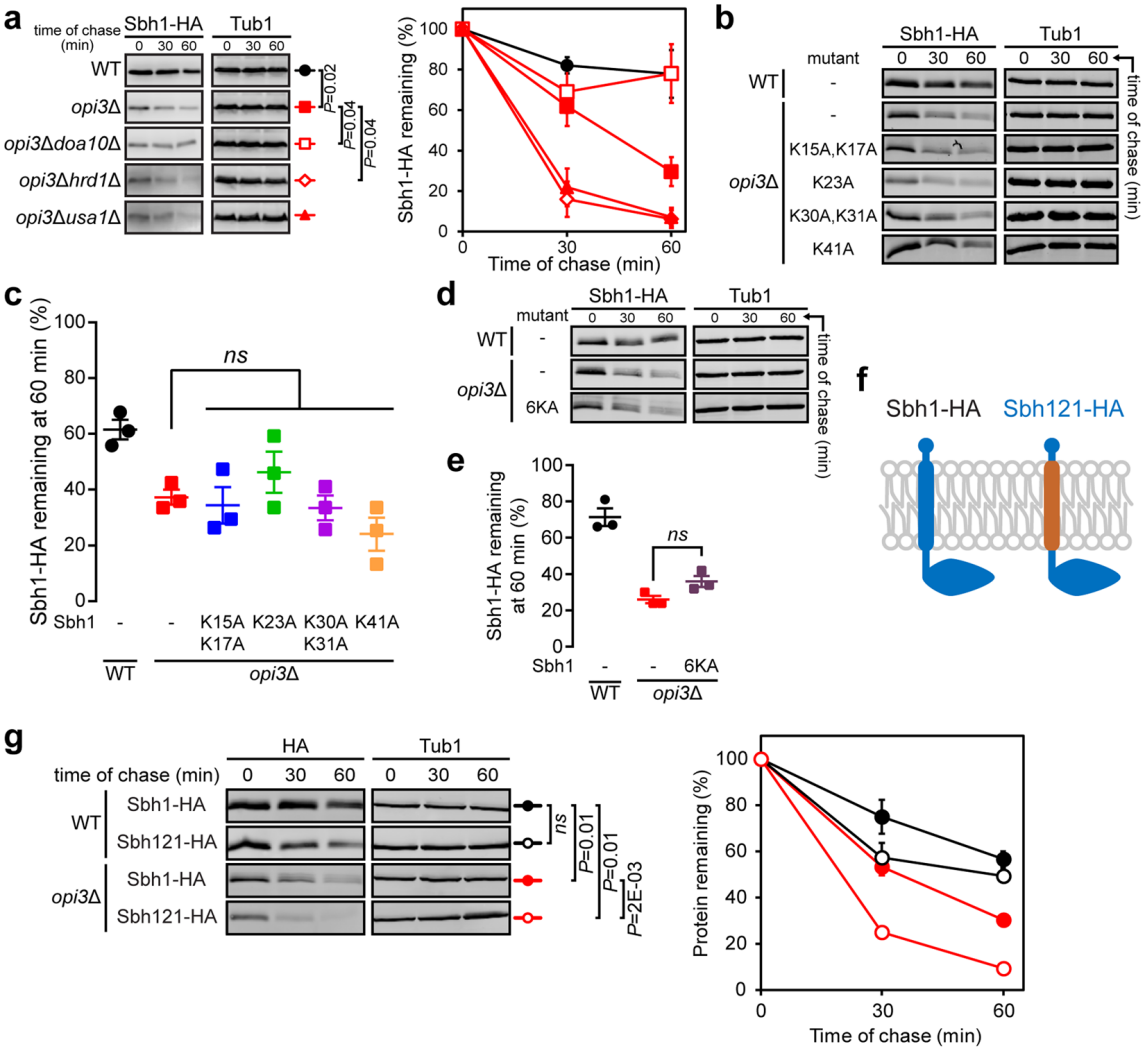
Sbh1 is destabilised from its transmembrane domain and degraded in a Doa10-dependent manner. (**a**) The degradation of Sbh1-HA was analysed in WT, *opi3*Δ, *opi3*Δ*doa10*Δ, *opi3*Δ*hrd1*Δ, and *opi3*Δ*usa1*Δ cells after blocking translation with cycloheximide. Proteins were separated by SDS-PAGE and detected by immunoblotting with antibodies against the HA tag and Tub1 as loading control. **(b)** The degradation of Sbh1-HA in WT and *opi3*Δ cells or HA-tagged Sbh1 cytosolic lysine mutants in *opi3*Δ cells treated as in (**a**). **(c)** Percentage Sbh1 remaining at the 60 min time point from (**b)**. **(d)** The degradation of Sbh1-HA in WT and *opi3*Δ cells or HA-tagged Sbh1 with all cytosolic lysine residues concurrently mutated to alanine [Sbh1(6KA)] in *opi3*Δ cells treated as in (**a**). **(e)** Sbh1 percentage remaining at the 60 min time point from (**d**). **(f)** Schematic diagram of the HA-tagged chimeric Sbh1 protein with its transmembrane domain replaced with that of Sbh2 (Sbh121-HA). The ER lumen and cytosol are at the top and bottom of the membrane, respectively. (**g**) The degradation of Sbh1-HA and Sbh121-HA in WT and *opi3*Δ cells treated as in (**a**). Data shown is the mean ± SEM (n = 3). All uncropped immunoblot images are included in the Supplementary File. Statistical analyses were subjected to paired two-tailed Student’s t-test. *ns*, non-significant.

To validate that Sbh1 is indeed degraded in a Doa10-dependent manner, we performed a cycloheximide chase assay to monitor Sbh1 stability in different ERAD mutants. Sbh1 was found to be fully stabilised in *opi3*Δ*doa10*Δ but not in *opi3*Δ*hrd1*Δ and *opi3*Δ*usa1*Δ mutants (Fig. 5a). Hrd1 and Usa1 are both part of the Hrd1 complex that recognises lesions within the luminal domains of membrane and soluble proteins (ERAD-L) and those found within transmembrane regions (ERAD-M)^70^. As some misfolded proteins in the ER are routed to the vacuole for degradation, we confirmed that Sbh1 degradation under lipid bilayer stress is independent of the vacuolar degradation pathway as shown by a similar degradation profile between *opi3*Δ and *opi3*Δ*pep4*Δ cells (Supplementary Fig. S4a). Conversely, Sbh1 degradation showed dependency on Cue1, a conserved element in both the Doa10 and Hrd1 complexes. Together with the MYTH screen results, it suggests that Sbh1 is exclusively targeted for degradation by the ERAD Doa10 complex.

To further elucidate how Sbh1 could be targeted for degradation by the Doa10 complex during lipid bilayer stress, we mutated Sbh1 cytosolic lysine residues to alanine individually [Sbh1(K15A,K17A), Sbh1(K23A), Sbh1(K30A,K31A), and Sbh1(K41A)] and in combination [Sbh1(6KA)]. The E3 ubiquitin-protein ligase Doa10 has been extensively reported to recognise ER proteins with cytosolic lesions resulting in the transfer of ubiquitin to lysine residues^62,71–77^. The degradation rates of Sbh1(K15A,K17A), Sbh1(K23A), Sbh1(K30A,K31A), and Sbh1(K41A) expressed in *opi3*Δ cells were similar to unmutated Sbh1 (Fig. 5b,c). Similarly, Sbh1(6KA) destabilisation was comparable to unmutated Sbh1 in *opi3*Δ cells (Fig. 5d,e). All Sbh1 lysine to alanine variants rescued the growth defect of *sbh1*Δ*sbh2*Δ and are therefore functional (Supplementary Fig. S4b). Together, these findings suggest that Sbh1 is targeted for degradation by the Doa10 complex independently from the ubiquitination of its cytosolic lysine residues. The yeast paralogue of Sbh1, Sbh2, is degraded by Doa10 through an intramembrane degron^50^. Thus, we examined the degradation of Sbh1 containing the transmembrane domain of Sbh2 in the *opi3*Δ mutant strain (Sbh121-HA, Fig. 5f,g). Surprisingly, the Sbh121-HA chimera did not exhibit an increased stability in *opi3*Δ cells and was instead further destabilised under lipid bilayer stress compared to Sbh1-HA. The transmembrane domains of Sbh1 and Sbh2 largely dictate their propensities for binding with their cognate complex subunits, while that of Sbh2 has additionally been identified to house a degron^37,50^. It is then likely that Sbh121-HA, having adapted the degradation mechanism and binding affinity of Sbh2, fails to interact with the Sec61 (or Ssh1) complex due to a dramatic change in its helical region, and is ultimately recognized rapidly by Doa10 for proteasomal destruction through the Sbh2 transmembrane degron. While cytosolic lysine residues were found to be dispensable for Doa10-mediated degradation of Sbh1 under lipid bilayer stress (Fig. 5b,c), the pronounced stability of HA-Sbh1 over Sbh1-HA in *opi3*Δ cells (Supplementary Fig. S3b) suggest that the cytosolic domain is critical for Sbh1 turnover. In support of this contention, the Sbh121 chimera tagged with HA on its N-terminal (HA-Sbh121) was highly stable under lipid bilayer stress (Supplementary Fig. S4c). Together, these findings suggest that the Doa10 complex recognises free Sbh1 that becomes increasingly accessible during lipid bilayer stress, perhaps due to the change in the ER membrane properties.

## Discussion

Upon ER stress, a subset of luminal and transmembrane ER-localised proteins is upregulated as part of the UPR programme. Previously, we demonstrated that some of these proteins are in low abundance while other components of their respective complexes are successfully upregulated by the UPR upon lipid bilayer stress, suggesting the premature degradation of proteins that are normally upregulated by the UPR^14^. The results presented in this study show that a subset of ER-resident transmembrane proteins, which form part of the UPR programme, are prematurely degraded in lipid-perturbed *opi3*Δ cells, potentially through extensive ER membrane remodelling and the corresponding changes in biophysical properties of the membrane. In this study, the Sec61 translocon complex component, Sbh1, is prematurely degraded by the Doa10 complex independently from its cytosolic lysine residues. While the Sec61 complex primarily functions in protein import into the ER, early studies in both yeast and *in vitro* mammalian cell models have postulated its involvement in retrotranslocation of misfolded proteins as part of the ERAD pathway. Mutations in yeast Sec61 caused the failure in the export of misfolded secretory proteins from the ER for destruction in the cytosol^78^, while the mammalian Sbh1 homologue, Sec61β, has been shown to directly associate with a client transmembrane protein *en route* to proteosomal degradation^79^. As a consequence of decreased Sbh1 levels, the protein quality control effector pathway under the UPR programme may also be directly compromised under lipid bilayer stress. Together, these suggest that lipid bilayer stress-induced premature degradation of transmembrane ER proteins affect a wide spectrum of ER functions and ultimately contribute to the development of chronic ER stress.

The proteostasis network undergoes extensive remodelling upon PC depletion in yeast^14^. Although a large subset of proteins is transcriptionally increased in these stressed cells, we observed that key proteins are rapidly degraded and are indeed sensitive to membrane phospholipid variations. Out of the 66 proteins, which displayed decreased protein abundance despite being genetically upregulated, 40% are transmembrane proteins. As 30% of the proteome is predicted to be either integral or peripheral membrane proteins^56^, transmembrane proteins are considerably more sensitive to lipid bilayer stress compared to other types of proteins. Furthermore, ER-resident proteins comprise a large proportion of the identified transmembrane proteins, thereby suggesting this organelle is more vulnerable to the effects of lipid bilayer stress, and this in turn affects transmembrane protein integrity in the ER.

Cylindrical PC generates minimal curvature while conical PE promotes negative membrane curvature^44,80,81^. The phospholipid intermediate MMPE, with physical properties similar to that of PE, becomes highly abundant under the ablation of *OPI3* (Fig. 1a). The virtual absence of sterol at the ER, a key regulator of membrane fluidity, could contribute to its susceptibility to changes in the biophysical properties of the membrane through lipid variation^82–84^. Additionally, the replacement of PC with MMPE contributes to the stiffening of the membrane^46^, in agreement with our data (Fig. 3c–e) and previous findings^47^. Preferably, fluidity of the ER membrane should be directly measured *in vivo*. However, technical limitations pose a challenge to this, especially when compared to the breadth of approaches available for characterising the fluidity of the plasma membrane^85–88^. Loss of protein functions and rapid degradation at the plasma membrane have been observed to correlate with changes in lipid bilayer properties, particularly the alteration of lipid rafts^89–91^. Thus, it is conceivable that Sbh1 is destabilised through a shift in its interactome and the subsequent increase in degron recognition by the Doa10 complex during lipid bilayer stress. However, other factors might partially contribute to Sbh1 instability due to the global effect of PC deficiency. Our MYTH screen results suggest the loss of Sbh1 interactors results in prolonged Sbh1 degron accessibility to the Doa10 complex, decreasing Sbh1 half-life. Similarly, the Sbh1 yeast paralogue, Sbh2, is rapidly degraded in a Doa10-dependent manner when dissociated of its partners^50^. Interestingly, none of the Sbh1 cytosolic lysine residues are required for its degradation through the Doa10 complex (Fig. 5b–e) suggesting Sbh1 could by atypically ubiquitylated as reported for the Doa10 substrate Asi2^92^. Sbh2 was similarly demonstrated to be degraded by the Doa10 complex independently of the ubiquitin-conjugating enzyme Ubc6, while Sbh2 lacking four adjacent lysine residues in its cytosolic domain was degraded in a Ubc6-dependent manner^93^.

**Figure 6:**
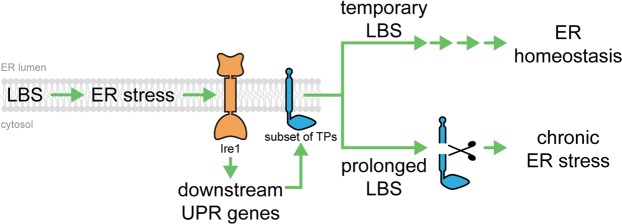
Premature degradation of transmembrane proteins leads to chronic ER stress. Under normal conditions, ER homeostasis can be reached from lipid bilayer stress (LBS) through the regulation of downstream UPR target genes. The UPR transactivator, Ire1, senses ER stress from the accumulation of misfolded proteins and/or LBS. However, under prolonged lipid bilayer stress, ER homeostasis could not be achieved due to the premature degradation of a subset of misfolded proteins (blue protein) leading to chronic ER stress and eventually to cell death.

The coordinated upregulation of the proteostasis network by the UPR serves as an important stress recovery mechanism that helps cells cope with the otherwise lethal effects of lipid bilayer stress^14^. Despite the robust stress response activation under lipid bilayer stress, the UPR programme fails to increase the expression levels of a subset of transmembrane proteins. The premature degradation of these transmembrane proteins can prevent an effective proteostatic response especially under prolonged lipid bilayer stress (Fig. 6). However, under prolonged lipid bilayer stress, cells fail to reach ER homeostasis with an insufficient pool of key ER proteins, leading to chronic ER stress. Consistent with this model, we have previously demonstrated that the UPR is essential to compensate for defects in protein translocation and ERAD during lipid bilayer stress, perhaps from a significant decrease of endogenous Sbh1 and Cue1, respectively^14^.

Altogether, our findings provide a causal mechanism to chronic ER stress in the context of lipid bilayer stress. Notably, a subset of ER-resident transmembrane proteins is prematurely degraded, preventing homeostasis within the ER. Further investigation on the β subunit of Sec61 ER translocation complex, Sbh1, is warranted to demonstrate the mechanism by which its degron becomes exposed due to a global loss of interacting partners under lipid bilayer stress and consequently results in premature degradation by the Doa10 complex.

## Methods

### Statistics

Error bars indicate standard error of the mean (SEM), calculated from at least three biological replicates, unless otherwise indicated. *P* values were calculated using two-tailed Student’s t test, unless otherwise indicated, and reported as *P* = value in figures.

### Strains and antibodies

*Saccharomyces cerevisiae* strains used in this study are listed in Supplementary Table S2. Strains were generated using standard cloning protocols. Anti-Kar2 polyclonal rabbit antibody and anti-Sec61 polyclonal rabbit antibody were gifts from Davis Ng (Temasek Life Sciences Laboratories, Singapore). Anti-HA mouse monoclonal antibody HA.11 (Covance), anti-Pgk1 mouse monoclonal antibody (Invitrogen), anti-GFP mouse monoclonal antibody (Roche) anti-tubulin mouse monoclonal antibody 12G10 (DHSB), anti-myc mouse monoclonal antibody (Invitrogen), anti-Flag mouse monoclonal antibody (Sigma), anti-LexA monoclonal mouse antibody (Santa Cruz Biotechnology) and anti-LexA polyclonal rabbit antibody (Abcam) were commercially purchased. Secondary antibodies goat anti-mouse IgG-DyLight 488 (Thermo Fisher, Waltham, MA), goat anti-rabbit IgG-DyLight 550 (Thermo Fisher), goat anti-mouse IgG-AlexaFluor488 (Invitrogen), goat anti-mouse IgG-HRP (Santa Cruz Biotechnology), goat anti-rabbit IgG-HRP (Santa Cruz), goat anti-mouse IgG-IRDye 800 (LI-COR Biosciences) and goat anti-rabbit IgG-IRDye 680 (LI-COR Biosciences) were commercially purchased.

### Plasmids used in this study

Plasmids and primers used in this study are listed in Supplementary Tables S3 and S4, respectively. Plasmids were constructed using standard cloning protocols. All coding sequences of constructs used in this study were sequenced in their entirety. The plasmid pJC835 containing the *HAC1*^*i*^ gene in pRS316 was previously described^15^. The plasmids pGT0179, pGT0181, pGT0183, and pGT0185, were generated by amplifying the promoter and open reading frame of *NSG2*, *CUE1*, *SBH1*, and *EMC4* with primer pairs BN033-034, BN029-030, BN035-036, and BN031-032, respectively, from the template WT genomic DNA (gDNA). PCR products of *NSG2, SBH1*, and *EMC4* were digested with the restriction enzymes *NotI* and *NcoI* before being ligated into the corresponding restriction sites in pRS315. *CUE1* PCR product was digested with the restriction enzymes *NcoI* and *PstI* before being ligated into the corresponding restriction sites in pRS315. The plasmid pGT0288 was generated by amplifying the open reading frame of Sbh1 with primer BN027 and BN028 from WT gDNA and digested with the restriction enzyme *SfiI* before being ligated into the corresponding restriction sites in pBT3N. The plasmid pGT0350 was generated by Gibson assembly to join the promoter and open reading frame of *SSS1* with primers BN013 and BN014 from WT gDNA with a 3 × Flag tag amplified with primers BN015 and BN016 from pGT0284 into pGT0001. Plasmids pGT0352, pGT0445, pGT0446, and pGT0447 were generated by performing site-directed mutagenesis on pGT0183 with primer pairs BN037-BN038, PS153-PS154, PS141-142, and PS143-144, respectively, as previously described^94^. The plasmid pGT0459 was generated by sequential site-directed mutagenesis from pGT0352 using primer pairs PS143-PS144, PS141-PS142, and PS139-140 as previously described^94^. The plasmid pGT0497 was generated by replacing the Sbh1-HA open reading frame present in pGT0183 with the coding sequence for HA-Sbh1 amplified from STK05-5-4 with primer pair PS201-PS202. The insert was introduced into a linearized vector amplified with primer pair PS199-PS200 from pGT0183 by Gibson Assembly. The plasmid pGT0459 was similarly generated by replacing the *SBH1* sequence in pGT0183 with the coding sequence for the chimeric *SBH121* amplified from STK05-8-5 with primer pair PS207-PS208. The insert was introduced into the linearized pGT183 vector amplified with primer pair PS205-PS206 through Gibson Assembly.

### Spotting growth assay

Strains were grown to saturation in appropriate selective medium overnight at 30 °C. Cultures were diluted to 0.15 OD_600_/ml and serially diluted fivefold for a total of six dilutions. Ten microliters of each dilution was spotted onto SC-LEU plates and incubated at 30 °C or 38 °C until the appearance of colonies.

### Indirect immunofluorescence

Indirect immunofluorescence was carried out as previously described^95^. Typically, cells were grown to early log phase at 30 °C in selective synthetic complete media, fixed in 3.7% formaldehyde and permeabilised. After blocking with 3% BSA, staining was performed using mouse anti-HA (1:200), mouse anti-LexA (1:50), rabbit anti-Kar2 (1:1,000), rabbit anti-LexA (1:500) or mouse anti-GFP (1:200) followed by goat anti-mouse IgG-Alexa Fluor 488 secondary antibody (1:2,000) and goat anti-rabbit IgG-DyLight 550 (1:2,000). Samples were visualised using a Zeiss LSM 710 microscope with a 100 × 1.4 NA oil Plan-Apochromat objective (Carl Zeiss MicroImaging).

### Cycloheximide chase assay

Cycloheximide chase assay was carried out as previously described^96^. Typically, 6 OD_600_ units of early log phase cells were grown in synthetic media. Protein synthesis was inhibited by adding 200 µg/ml cycloheximide. Samples were taken at designated time points. Cell lysates from these samples were resolved by SDS-PAGE and transferred onto a nitrocellulose membrane. Immunoblotting was performed with appropriate primary antibodies and horseradish peroxidase-conjugated secondary antibodies or IRDye-conjugated secondary antibodies. Proteins were visualised using the ECL system (C-DiGit Chemiluminescent Western Blot Scanner) or the NIR fluorescence system (Odyssey CLx Imaging System). Values for each time point were normalised using anti-Pgk1 or anti-Tub1 as loading controls. Tonal quality was adjusted for representative images through ImageStudio Lite Version 5.2 (LI-COR Biosciences) where appropriate and was followed by quantification. All comparative analyses were done on immunoblots performed in parallel using samples derived from the same experiment. Uncropped immunoblot images are included as a Supplementary File.

For quantification of protein abundance by indirect immunofluorescence following attenuation of protein translation, early log phase cultures were treated with 200 µg/ml cycloheximide, and aliquots were taken at zero- and 60-min time points. Samples were fixed, permeabilised and incubated with the appropriate antibodies as described earlier. Mounted cells were visualised using a Zeiss LSM 710 microscope with a 100 × 1.4 NA oil Plan-Apochromat objective (Carl Zeiss MicroImaging). Stack images of 11 optical sections spaced 0.3 µm apart were taken and used to generate maximum intensity projection images. Background-subtracted fluorescent signals for the protein of interest were quantified using Fiji imaging software and normalised against Kar2.

### Alkaline carbonate extraction

Alkaline carbonate extraction was carried out as previously described^97^. Five OD_600_ units of early log phase cells were resuspended in 1.2 ml of 10 mM sodium phosphate pH 7.0, 1 mM PMSF and protease inhibitor cocktail (PIC). An equal volume of 0.2 M NaHCO_3_ (pH 11.5) was added to cell lysates incubated 30 min at 4 °C and spun down at 100,000 × *g* for 30 min, 4 °C. The pellet (membrane fraction) was solubilised in 3% SDS, 100 mM Tris, pH 7.4, 3 mM DTT and incubated at 100 °C for 10 min. Proteins from total cell lysate and supernatant fractions (collected from centrifuged lysate) were precipitated with 10% trichloroacetic acid (TCA) and spun down 30 min at 18,400 × *g*, 4 °C. Proteins were resuspended in TCA resuspension buffer (100 mM Tris, pH 11.0, 3% SDS).

### Proteinase K digestion assay

Fifty OD_600_ units of early log phase cells were pelleted and resuspended in 1 ml Tris buffer (50 mM Tris pH 7.4, 50 mM NaCl, 10% glycerol, 1 mM PMSF and PIC). Cells were mechanically disrupted 10 times using 0.5 mm zirconium beads with a vortex mixer at maximum speed for 60 s each, with 5 min incubation on ice between intervals. The supernatant was collected by spinning down the lysate at 800 × *g* for 5 min at 4 °C. The clarified cell lysate was spun down at 100,000 × *g* for 1 h at 4 °C. The pellet was resuspended and washed with 0.5 ml Tris buffer without PMSF and PIC. Approximately 5 OD_600_ equivalent of microsomes were incubated with 1 mg/ml Proteinase K (Promega, Fitchburg, WI) and 1% Nonidet P40 substitute (Sigma-Aldrich) when indicated and incubated at 37 °C for 30 min. To quench the reaction, 5 mM PMSF was added followed by TCA precipitation. Samples were resolved by SDS-PAGE and transferred onto a nitrocellulose membrane. Immunodetection was performed with appropriate primary antibodies and IRDye-conjugated secondary antibodies. Immunoreactive species were visualised using the NIR fluorescence system (Odyssey CLx Imaging System).

### Lipid extraction and fatty acid analysis

For whole cells, 10 OD_600_ of early log phase cells were pelleted, washed and resuspended in 1 ml ice-cold water. A 100 µl aliquot was taken, mechanically lysed and quantified for total protein through bicinchoninic acid (BCA) protein quantification (Sigma-Aldrich). Equal volumes of cell lysates were lyophilised using Virtis Freeze Dryer under vacuum. For lipid extraction of microsomes, 50 OD_600_ of early log phase cells were pelleted, washed with ice-cold water and resuspended in 1 ml of Tris buffer (50 mM Tris-HCl pH 8.0, 150 mM NaCl, 5 mM EDTA pH 8.0, 1 mM PMSF and PIC). Cells were mechanically disrupted 15 times at 30 s intervals using 0.5 mm zirconium beads at maximum speed of a vortex mixer at 4 °C. The supernatant was collected by spinning down the lysate at 800 × *g* for 5 min at 4 °C. The clarified lysate was spun down at 100,000 × *g* for 1 h at 4 °C. The pellet was resuspended in 100 µl ddH_2_O and sonicated for 30 min. A 10 µl aliquot was taken, solubilized, and measured for protein concentration with BCA protein quantification. Equal volumes of homogenized microsomes were lyophilized using Virtis Freeze Dryer under vacuum. Lyophilised samples were added with 100 µl of 1 mM pentadecanoic acid (C15:0) and 300 µl of 1.25 M HCl-MeOH (Sigma-Aldrich) and incubated at 80 °C for 1 h for hydrolysis and esterification of FAs into FA methyl esters (FAME). FAMEs were extracted three times with 1 ml of hexane. Combined extracts were concentrated and separated on a gas chromatography with flame ionization detector (GC-FID; GC-2014; Shimadzu) equipped with an Ulbon HR-SS-10 capillary column (nitrile silicone, 50 m × 0.25 mm; Shinwa Chemical Industries). FAs were identified with reference to Supelco 37 component FAME mix (Sigma-Aldrich). FA concentrations were normalised to protein content and reported as absolute values relative to the C15:0 internal standard.

### Fluorescence recovery after photobleaching

The fluorescence recovery after photobleaching (FRAP) experiment was carried out as previously described^45^. Typically, early log phase cells expressing Sec63-sfGFP were fixed on concanavalin A-treated coverslips fitted in Attofluor cell chambers (Thermo Fisher). Cells were imaged for 5 s followed by photobleaching a region of interest of 82 × 82 pixels at 100% intensity of the 488 nm laser under 5 × magnification. Subsequently, images were taken at 1.57 s intervals for a total of 160 s. Images were acquired using a Zeiss LSM 710 microscope with a 100 × 1.4 NA oil Plan-Apochromat objective (Carl Zeiss MicroImaging) and optical sections of 4.2 μm. ZEN software (Zeiss, Oberkochen) was used for image acquisition and analysis. Magnification, laser power, and detector gains were identical across samples. For data analysis, the fluorescence intensities of three regions of interest were measured for the duration of the experiment: the region of interest (ROI), a region outside of the cell to measure background fluorescence (BG), and a non-photobleached region within the cell were monitored to measure the overall photobleaching and fluorescence variation (REF). Normalised fluorescence intensities [F(*t*)_*norm*_] were calculated for each time point using Eq. 1 ^98^. F(i) denotes the initial fluorescence intensities.

1 $$F{(t)}_{norm}=\frac{F{(t)}_{ROI}-{F}_{BG}}{F{(t)}_{REF}-{F}_{BG}}\times \frac{F{(i)}_{REF}-{F}_{BG}}{F{(i)}_{ROI}-{F}_{BG}}$$

Fluorescent recovery was analysed by calculating half maximal fluorescence intensity (t_½_) using Eq. 2 ^99^. F_0_ denotes the normalised initial fluorescence intensity, F_∞_ the normalised maximum fluorescence intensity and F(t) the normalised fluorescent intensity at each time point.

2 $$F(t)=\frac{{F}_{0}+{F}_{\infty }\frac{t}{{t}_{1/2}}}{1+\frac{t}{{t}_{1/2}}}$$

The t_½_ values were plotted using GraphPad Prism 5.0.

### Membrane yeast two-hybrid system screen

The membrane yeast two-hybrid (MYTH) screen was carried out as previously described^48^. MYTH utilises the split ubiquitin moieties namely, the N-terminus (N_ub_) and C-terminus (C_ub_). Briefly, the MYTH bait was generated by fusing the C_ub_-LexA-VP16 tag at the N-terminal of Sbh1 (TF-C_ub_-Sbh1) under the control of the promoter *CYC1* and transformation into the NMY51 yeast reporter strain. TF-C_ub_-Sbh1 protein localization was verified by indirect immunofluorescence using anti-LexA antibodies. Seven micrograms of N_ub_G-X cDNA prey library (Dualsystems) was transformed into 35 OD_600_ units of reporter cells. Interactors were isolated on selective complete (SC) media lacking tryptophan, leucine, adenine and histidine complemented with 80 µg/mL X-Gal and 5 mM 3-amino-1,2,4-triazole (3-AT) and grown for two days at 30 °C. The histidine inhibitor 3-AT was used to reduce false positive colonies. Only colonies which displayed robust growth on selective media and the formation of a blue colour were selected for further analysis. The prey cDNA plasmids were isolated and identified through sequencing. The list of interactors was verified through a bait dependency test, wherein all identified interactors are retransformed back into the original bait strain, together with a non-specific negative control bait construct encoding for the single-pass transmembrane domain of the human T-cell surface glycoprotein CD4 tagged to C_ub_-LexA-VP16^54^. Interactors that activated the reporter system in yeast carrying the negative control bait were removed from the list of interactors. Yeast that harbour the prey and the bait-of-interest and did not display the requisite growth phenotype were likewise removed from the list of interactors.

### Co-immunoprecipitation

Native lysis protocol was carried out as previously described^100^. Briefly, 40 OD_600_ units of exponentially growing early log phase cells were harvested and resuspended in 1 ml native lysis buffer (50 mM Tris, pH 7.5, 150 mM NaCl, 5 mM EDTA, 1 mM PIC and 1 mM PMSF). Cells were mechanically disrupted 10 times using 0.5 mm zirconium beads with a vortex mixer at maximum speed for 60 s each, with 5 min incubation on ice between intervals. The supernatant was collected by spinning down the lysate at 800 × *g* for 5 min at 4 °C. The clarified cell lysate was spun down at 100,000 × *g* for 1 h at 4 °C. The pellet was solubilised in native lysis buffer with 1% digitonin (Calbiochem) 1 h at 4 °C. The resulting lysate was cleared by centrifugation at 16,000 × *g* for 10 min, 4 °C prior to immunoprecipitation. Solubilised microsomes were incubated with Protein G beads and anti-HA antibodies overnight at 4 °C. Beads were washed thrice lysis buffer containing 0.5% digitonin and twice with TBS. Proteins were separated using SDS-PAGE and visualised by immunoblotting as described above.

### β-galactosidase reporter assay

The β-galactosidase reporter assay was carried out as previously described^17^. Typically, cells are grown to early log phase, and tunicamycin was added to growth cultures when necessary at a concentration of 2.5 μg/ml to cells 1 h prior to harvest to induce UPR activation. Four OD_600_ units of cells were collected and resuspended in 75 μl LacZ buffer (125 mM sodium phosphate, pH 7, 10 mM KCl, 1 mM MgSO_4_, 50 mM β-mercaptoethanol). An aliquot of 25 μl was transferred into 975 μl ddH_2_O and the absorbance was measured at 600 nm. To the remaining resuspension, 50 μl chloroform and 20 μl 0.1% SDS were added and the resulting mixture was vortexed vigorously for 20 s. The reaction was started with the adding 700 μl of 2 mg/ml ONPG (2-nitrophenyl-*D*-galactopyranoside; Sigma) in LacZ buffer. Then, the reaction was quenched with 500 μl of 1 M Na_2_CO_3_, and total reaction time was recorded. Samples are spun for 1 min at maximum speed. Absorbance of the resulting supernatant was measured at 420 nm and 550 nm. The β-galactosidase activity was calculated using Eq. (3).

3 $${\rm{Miller}}\,{\rm{units}}=1000\times ({{\rm{OD}}}_{420}-1.75\times \,{{\rm{OD}}}_{550})/({\rm{t}}\times ({\rm{VA}}/{\rm{VR}})\times {{\rm{OD}}}_{600})$$

The values were then normalised to the activity of WT.

## Supplementary information

SREP-19-02941 Supplementary Information

Table S1
