# Supplementary material for: Membrane phospholipid alteration causes chronic ER stress through early degradation of homeostatic ER-resident proteins
Source: Sci Rep. 2019 Jun 14;9:8637. doi: 10.1038/s41598-019-45020-6 (PMC6572771; doi:10.1038/s41598-019-45020-6)
Supplement: Supplementary file 1 — SREP-19-02941 Supplementary Information [file 41598_2019_45020_MOESM1_ESM.pdf]

Membrane phospholipid alteration causes chronic ER stress through early degradation of homeostatic ER-resident proteins

Peter Jr. Shyu[1], Benjamin S.H. Ng[1], Nurulain Ho, Ruijie Chaw, Yi Ling Seah, Charlie Marvalim, Guillaume Thibault

School of Biological Sciences, Nanyang Technological University, Singapore, 637551

[1]These authors have contributed equally to this work

Correspondence to:
Guillaume Thibault, Tel: +65 6592 1787; email: thibault@ntu.edu.sg

SUPPLEMENTARY FILES

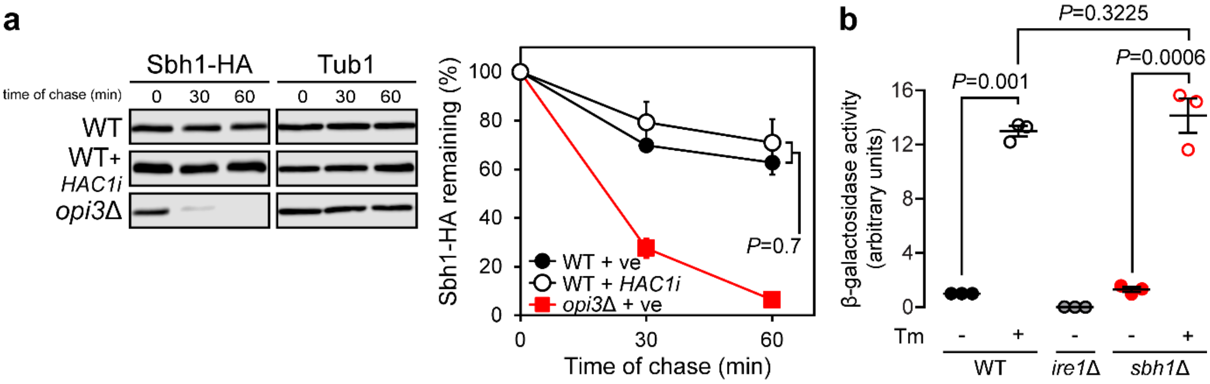

Figure S1. Strong activation of the UPR does not destabilise Sbh1. (a) The degradation of Sbh1-HA was analysed in WT and *opi3Δ* cells containing control vector (ve) or *HAC1^{i}*-bearing plasmid after blocking translation with cycloheximide. Proteins were separated by SDS-PAGE and detected by immunoblotting with antibodies against the HA tag and Tub1 as loading control. **(b)** Cells were grown to early log phase at 30˚C in selective synthetic complete media. UPR induction was measured using a *UPRE-LacZ* reporter assay. Tm, tunicamycin. Data shown is the mean ± SEM (n = 3). All uncropped immunoblot images are included in the Supplementary File. Statistical analyses were subjected to paired two-tailed Student's t-test.

aaa

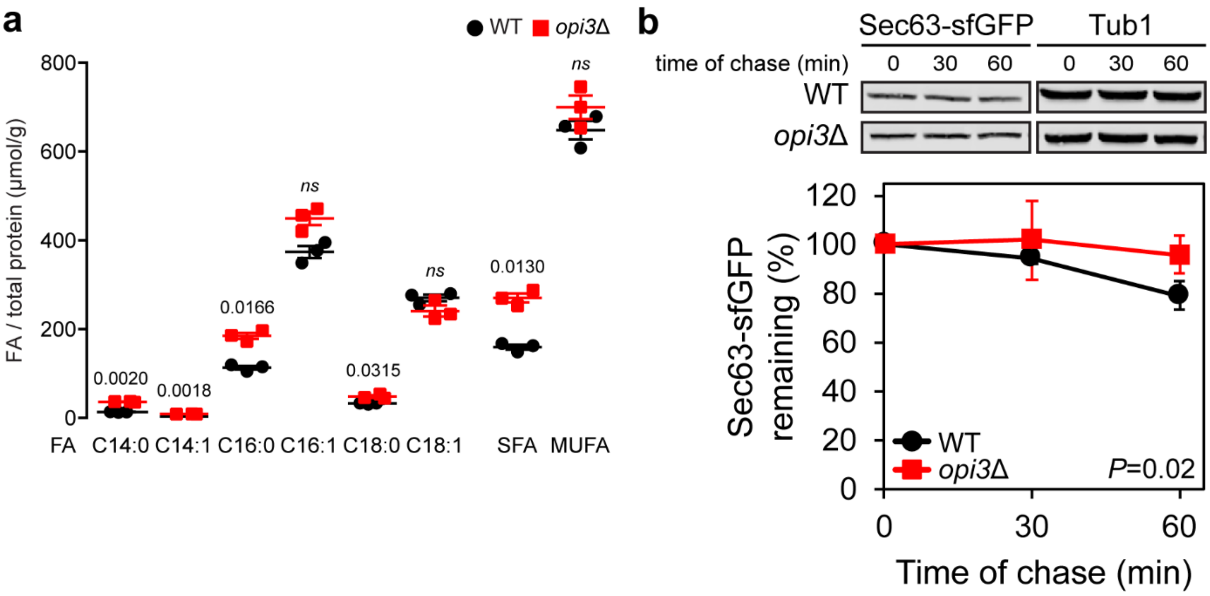

Figure S2. Sec63-sfGFP remains stable during fatty acid remodelling. (a) The abundance of different fatty acids (FA) species in WT and *opi3Δ* cell extracts were quantified by gas chromatography with FAME derivatisation. FAs are categorised based on chain length and degree of saturation. SFA, saturated fatty acid; MUFA, monounsaturated fatty acid. **(b)** The degradation of Sec63-sGFP was analysed in WT and *opi3Δ* cells after blocking translation with cycloheximide. Proteins were separated by SDS-PAGE and detected by immunoblotting with antibodies against the GFP epitope and Tub1 as loading control. Data shown is the mean ± SEM (n = 3). All uncropped immunoblot images are included in the Supplementary File. Statistical analyses were subjected to paired two-tailed Student's t-test.

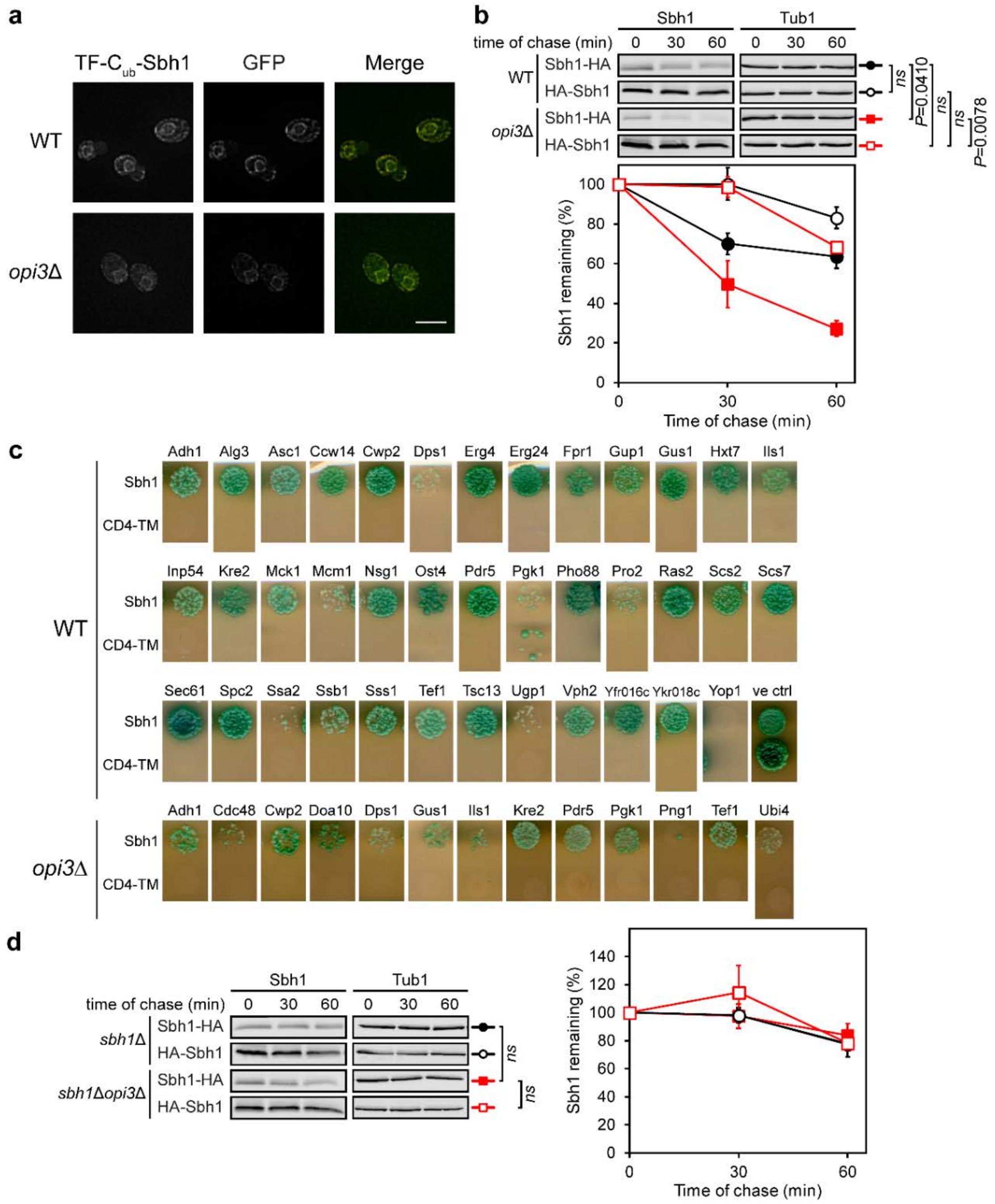

Figure S3. Validation of Sbh1 MYTH bait protein and interacting partners. (a) N-terminal reporter tagged Sbh1 (TF-C_{ub}-Sbh1) remains localised to the ER membrane in both WT and *opi3*Δ cells. Protein candidates were detected using antibodies against LexA and eroGFP as ER marker. Scale bar, 5 µm. **(b)** The degradation of N- and C-terminal HA-tagged variants of Sbh1 were analysed in WT and *opi3*Δ cells after blocking translation with cycloheximide. Proteins were separated by SDS-PAGE and detected by immunoblotting with antibodies against the HA tag and Tub1 as loading control. **(c)** Plasmids encoding for interactors of TF-C_{ub}-Sbh1 were retransformed into the bait strain and a negative control strain expressing the single-pass transmembrane domain of human T-cell surface glycoprotein CD4 fused to C_{ub}-LexA-VP16. The pOST1-N_{ub}I prey construct (ve ctrl) was used as positive control. **(d)** The degradation of N- and C-terminal HA-tagged variants of Sbh1 were analysed in *sbh1*Δ and *sbh1*Δ*opi3*Δ cells after blocking translation with cycloheximide. Proteins were separated by SDS-PAGE and detected by immunoblotting with antibodies against the HA tag and Tub1 as loading control. Data shown is the mean ± SEM (n = 3). All uncropped immunoblot images are included in the Supplementary File. Statistical analyses were subjected to paired two-tailed Student's t-test.

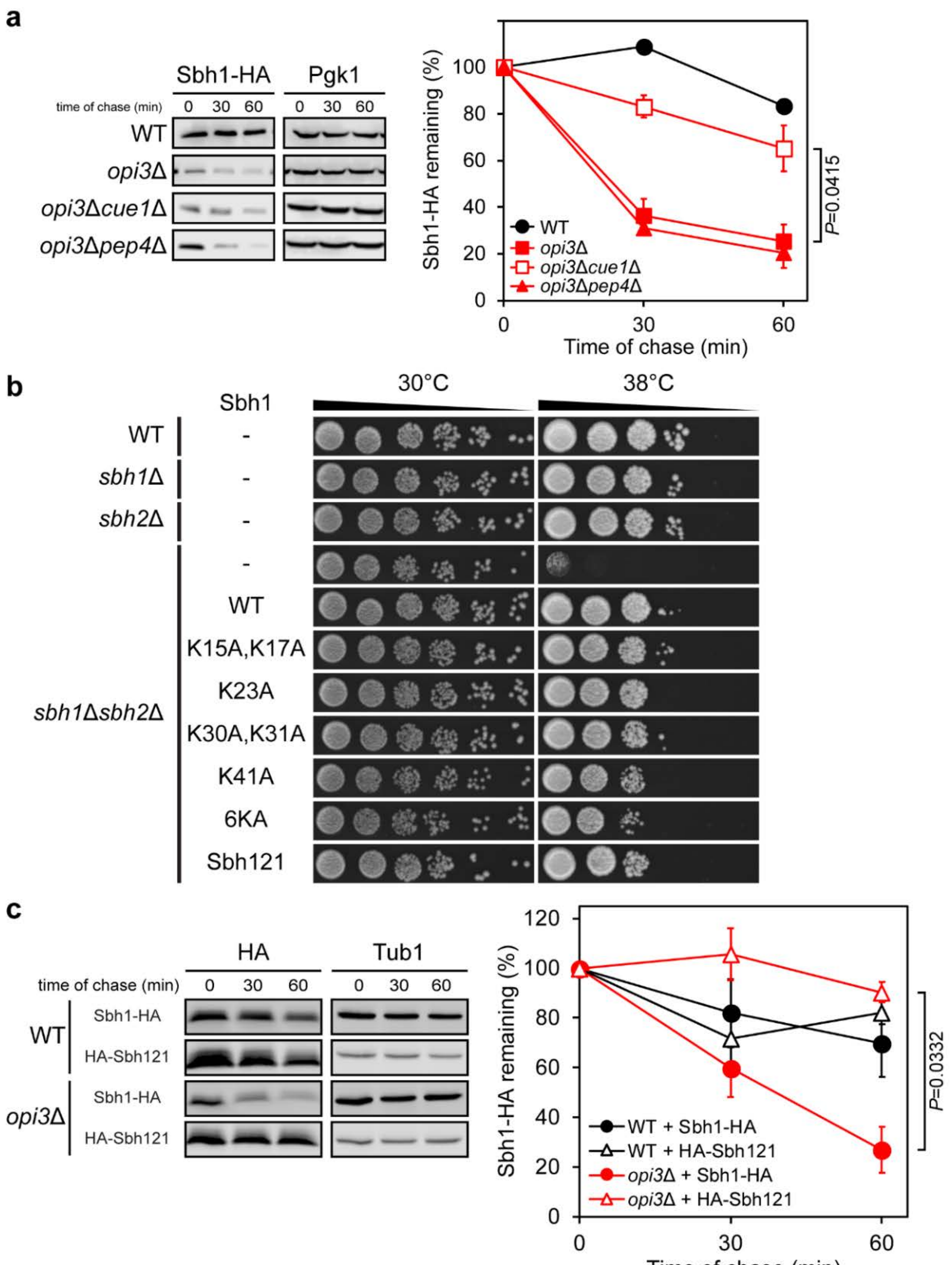

Figure S4. Stability and functionality of Sbh1 variants. (a) Sbh1 is degraded by ERAD and not the vacuolar pathway. The degradation of Sbh1-HA was analysed in WT, *opi3*Δ, *opi3*Δ*cue1*Δ, and *opi3*Δ*pep4*Δ cells after blocking translation with cycloheximide. Proteins were separated by SDS-PAGE and detected by immunoblotting with antibodies against the HA tag and PGK1 as loading control. **(b)** Strains harbouring vector controls or plasmids encoding C-terminal HA-tagged mutants of Sbh1 were grown to saturation at 30°C, and serial dilutions of the culture were spotted onto plates and further incubated at the indicated temperatures until the appearance of colonies. **(c)** The degradation of Sbh1-HA and HA-Sbh121 were analysed in WT and *opi3*Δ cells after blocking translation with cycloheximide. Proteins were separated by SDS-PAGE and detected by immunoblotting with antibodies against the HA tag and Tub1 as loading control. Data shown is the mean ± SEM (n = 3). All uncropped immunoblot images are included in the Supplementary File. Statistical analysis was subjected to paired two-tailed Student’s t-test.

Table S2. Strains used in the study

Strains	Genotype	Source
W303a	*MAT*a*, leu2-3,112, his3-11, trp1-1, ura3-1, can1-100, ade2-1*	[1]
GTY68	*MATa, opi3::KANMX,* W303 background	[2]
SKY309	*MAT*α, *prc1-1, SEC61myc9::TrpMX,* W303 background	[3]
YGT0315	*MAT*a, pGT0181, W303 background	This study
YGT0317	*MAT*a, *opi3::KANMX*, pGT0181, W303 background	This study
YGT0318	*MAT*a, pGT0182, W303 background	This study
YGT0320	*MAT*a, *opi3::KANMX*, pGT0182, W303 background	This study
YGT0321	*MAT*a, pGT0179, W303 background	This study
YGT0323	*MAT*a, *opi3::KANMX*, pGT0179, W303 background	This study
YGT0327	*MAT*a, pGT0185, W303 background	This study
YGT0329	*MAT*a, *opi3::KANMX*, pGT0185, W303 background	This study
YGT0330	*MAT*a, pGT0178, W303 background	This study
YGT0332	*MAT*a, *opi3::KANMX*, pGT0178, W303 background	This study
YGT0374	*MAT*a, pGT0183, W303 background	This study
YGT0375	*MAT*a, *opi3::KANMX*, pGT0183, W303 background	This study
YGT0432	*MAT*a, pJC835, W303 background	This study
YGT0540	*MAT*a, pGT0288, NMY51 background (*his3Δ200*, *trp-901*, *leu2-3,112*, *ade2*, *LYS::(lexAop)4-HIS3*, *ura3::(lexAop)8-LACZ*, *(lexAop)8-ADE2*, *GAL4*)	This study
YGT0541	MATa, *opi3::KANMX*, pGT0183, NMY51 background	This study
YGT0574	*MAT*a, *doa10::KANMX*, *opi3::KANMX*, pGT0183, W303 background	This study
YGT0575	*MAT*a, *hrd1::KANMX*, *opi3::KANMX*, pGT0183, W303 background	This study
YGT0576	*MAT*a, *usa11::KANMX*, *opi3::KANMX*, pGT0183, W303 background	This study
YGT0577	*MAT*a, pGT0003, W303 background	This study
YGT0671	*MAT*a, pGT0352, W303 background	This study
YGT0672	*MAT*a, *opi3::KANMX*, pGT0352, W303 background	This study
YGT0673	*MAT*a, pGT0183, pGT0001, W303 background	This study
YGT0674	*MAT*a, *opi3::KANMX*, pGT0183, pGT0001, W303 background	This study
YGT0690	*MAT*a, pGT0180 , W303 background	This study
YGT0691	*MAT*a, *opi3::KANMX*, pGT0180, W303 background	This study
YGT0721	*MAT*a, pGT0350, pGT0183, W303 background	This study
YGT0722	*MAT*a, *opi3::KANMX*, pGT0350, pGT0183, W303 background	This study
YGT0725	*MAT*a, pGT0350, pRS315, W303 background	This study
YGT0726	*MAT*a, *opi3::KANMX*, pGT0350, pRS315, W303 background	This study
YGT0761	*MAT*a, pGT0362, NMY51 background	This study
YGT0769	*MAT*a, pGT0366, W303 background	This study
YGT0770	*MAT*a, *opi3::KANMX*, pGT0366, W303 background	This study
YGT0771	*MAT*a, pGT0368, W303 background	This study
YGT0772	*MAT*a, *opi3::KANMX*, pGT0368, W303 background	This study
YGT0773	*MAT*a, pGT0365, W303 background	This study
YGT0774	*MAT*a, *opi3::KANMX*, pGT0365, W303 background	This study
YGT0874	*MAT*a, pPS1622, pGT0001, W303 background	This study
YGT0875	*MAT*a, *opi3::KANMX*, pPS1622, pGT0001, W303 background	This study
YGT1122	*MAT*a, pGT0445, W303 background	This study
YGT1123	*MAT*a, *opi3::KANMX*, pGT0445, W303 background	This study
YGT1124	*MAT*a, pGT0446, W303 background	This study
YGT1125	*MAT*a, *opi3::KANMX*, pGT0446, W303 background	This study
YGT1126	*MAT*a, pGT0447, W303 background	This study
YGT1127	*MAT*a, *opi3::KANMX*, pGT0447, W303 background	This study
YGT1148	*MAT*a, pGT0459, W303 background	This study
YGT1149	*MAT*a, *opi3::KANMX*, pGT0459, W303 background	This study
YGT1167	*MAT*a, STK05-5-9, W303 background	This study
YGT1168	*MAT*a, *opi3::KANMX*, STK05-5-9, W303 background	This study
YGT1169	*MAT*a, STK05-8-5, W303 background	This study
YGT1170	*MAT*a, *opi3::KANMX*, STK05-8-5, W303 background	This study
YGT1185	*MAT*α, *prc1-1, SEC61myc9::TrpMX, opi3::KANMX*, W303 background	This study

YGT1186	*MAT*a, pGT0497, W303 background	This study
YGT1187	*MAT*a, *opi3::KANMX*, pGT0497, W303 background	This study
YGT1190	*MAT*α, *sbh1::KANMX*, pGT0003, W303 background	This study
YGT1191	*MAT*a, *sbh2::KANMX*, pGT0003, W303 background	This study
YGT1192	*MAT*a, *sbh1::KANMX*, *sbh2::KANMX*, pGT0183, W303 background	This study
YGT1193	*MAT*a, *sbh1::KANMX*, *sbh2::KANMX*, pGT0497, W303 background	This study
YGT1194	*MAT*a, *sbh1::KANMX*, *sbh2::KANMX*, pGT0288, W303 background	This study
YGT1195	*MAT*a, *sbh1::KANMX*, *sbh2::KANMX*, pGT0352, W303 background	This study
YGT1196	*MAT*a, *sbh1::KANMX*, *sbh2::KANMX*, pGT0445, W303 background	This study
YGT1197	*MAT*a, *sbh1::KANMX*, *sbh2::KANMX*, pGT0446, W303 background	This study
YGT1198	*MAT*a, *sbh1::KANMX*, *sbh2::KANMX*, pGT0447, W303 background	This study
YGT1199	*MAT*a, *sbh1::KANMX*, *sbh2::KANMX*, pGT0459, W303 background	This study
YGT1203	*MAT*a, *sbh1::KANMX*, *sbh2::KANMX*, pGT0003, W303 background	This study
YGT1213	*MAT*α, *prc1-1, SEC61myc9::TrpMX,* pGT0003, pGT0350, W303 background	This study
YGT1214	*MAT*α, *prc1-1, SEC61myc9::TrpMX, opi3::KANMX*, pGT0003, pGT0350, W303 background	This study
YGT1215	*MAT*α, *prc1-1, SEC61myc9::TrpMX,* pGT0183, pGT0350, W303 background	This study
YGT1216	*MAT*α, *prc1-1, SEC61myc9::TrpMX, opi3::KANMX*, pGT0183, pGT0350, W303 background	This study
YGT1221	*MAT*a, *sbh1::KANMX*, pGT0183, W303 background	This study
YGT1222	*MAT*a, *sbh1::KANMX*, *opi3::KANMX,* pGT0183, W303 background	This study
YGT1223	*MAT*a, *sbh1::KANMX*, pGT0497, W303 background	This study
YGT1224	*MAT*a, *sbh1::KANMX*, *opi3::KANMX,* pGT0497, W303 background	This study
YGT1225	*MAT*a, pGT0527, W303 background	This study
YGT1226	*MAT*a, *opi3::KANMX,* pGT0527, W303 background	This study
YGT1227	*MAT*a, *sbh1::KANMX*, *sbh2::KANMX*, pGT0527, W303 background	This study

Table S3. Plasmids used in the study

Plasmid	Encoded protein	Promoter	Vector	Source
pJC31	β-galactosidase	*UPRE-CYC1*	pRS315	[4]
pPS1622	Sec63-sGFP	*SEC63*	pRS316	[5]
pJC835	Hac1	*HAC1*	pRS313	[1]
pPM28	eroGFP	*GAP*	pRS316	[6]
STK05-5-4	HA-Sbh1	*MET25*	p413MET25	[3]
STK05-8-5	HA-Sbh121	*MET25*	p413MET25	[7]
pGT0001	-	-	pRS313	[8]
pGT0003	-	-	pRS315	[8]
pGT0178	Coy1-HA	*COY1*	pRS315	This study
pGT0179	Nsg2-HA	*NSG2*	pRS315	This study
pGT0180	Scs7-HA	*SCS7*	pRS315	This study
pGT0181	Cue1-HA	*CUE1*	pRS315	This study
pGT0182	Erp5-HA	*ERP5*	pRS315	This study
pGT0183	Sbh1-HA	*SBH1*	pRS315	This study
pGT0185	Emc4-HA	*EMC4*	pRS315	This study
pGT0284	IRE1-3X FLAG	*IRE1*	pRS426	[9]
pGT0288	C_{ub}-LexA-VP16-Sbh1	*CYC1*	pBT3-N	This study
pGT0350	Sss1-3XFlag	*SSS1*	pRS313	This study
pGT0352	Sbh1(K41A)-HA	*SBH1*	pRS315	This study
pGT0362	CD4-C_{ub}-LexA-VP16	*CYC1*	pCMBV	[10]
pGT0365	Prm5-HA	*PRM5*	pRS315	This study
pGT0367	Yet3-HA	*YET3*	pRS315	This study
pGT0445	Sbh1(K15/17A)-HA	*SBH1*	pRS315	This study
pGT0446	Sbh1(K23A)-HA	*SBH1*	pRS315	This study
pGT0447	Sbh1(K30/31)-HA	*SBH1*	pRS315	This study
pGT0459	Sbh1(6KA)-HA	*SBH1*	pRS315	This study
pGT0497	HA-Sbh1	*SBH1*	pRS315	This study
pGT0527	Sbh121-HA	*SBH1*	pRS315	This study

Table S4. **Oligonucleotide primers used in the study**

Primer	Sequence (5' to 3')
BN013	CCGCGGTGGCGGCCGCCACTAGCCGATGTTATC
BN014	GTAGTCCGCATGCCCAACAATAACGTATCTGATTGG
BN015	GGGCATGCGGACTACAAAGACCATGACG
BN016	CGGCCGCCACCGCGGTGG
BN027	ATGAGGGCCATTACGGCCATGTCAAGCCCAACTCCTCC
BN028	CTCATGGCCGAGGCGGCCTTAAAATAACTTACCGGCAACTTTAGAAATAACATG
BN029	CTCATGCTGCAGATGGAGGATTCGAGATTGCTTATCACTTTG
BN030	CTCATGCCATGGGAGTCAGCAAACTTTGCAAATCTTTATCAC
BN031	AACGTCGCGGCCGCAGCAAATGATTCCTCGACTGAATATAAAGG
BN032	CCATGGCGCGCTAATCGGAAAACCATTGTAATCCATTATTATAATGAGCA
BN033	CTCATGCTGCAGATGGCCAATAGAGGAGAACCGG
BN034	CTCATGCCATGGGATGAGAATATAGATATCTTCCTAGTTTTCCAAACATTAG
BN035	CTCATGCTGCAGATGTCAAGCCCAACTCCTCC
BN036	CTCATGCCATGGGAAATAACTTACCGGCAACTTTAGAAATAACATG
BN037	AATTCGATTTTGGCGATTTATTCTGAT
BN038	ATTGCTGTTCGTGTTTTTCTTTGGAGC
PS139	TACTTTGCAAGCGAGAGCACAGGGAAGTTC
PS140	CGTTGACCACCTGGAGGAGTTGGG
PS141	AAGTTCACAAGCAGTTGCGGCAT
PS142	CCCTGTTTTCTCTTTTGCAAAGTACG
PS143	ATCCGCTCCAGCGGCAAACACGAACA
PS144	GCCGCAACTTTTTGTGAACTTCCCTGTTTT
PS153	AAAGTTGCGGCATCCGCTC
PS154	TTGTGAACTTCCCTGTGCTCTCGCTTGCAAAG
PS199	TTAATTAAGATCCGGCTCTAGATAATCTCTGC
PS200	TATGGAGTATGGTAGGAGGTAG
PS201	ACCTCCTACCATACTCCATATCTAGAACTAGTATGGCATAC
PS202	TAGAGCCGGATCTTAATTAATTAAAATAACTTACCGGC
PS205	GCCATGGCCTACCCATATGATGTTCCAG
PS206	TATGGAGTATGGTAGGAGGTAGAGTGTGG
PS207	TCCTACCATACTCCATAATGTCAAGCCCAACTCCTC
PS208	TGGGTAGGCCATGGCAAATAACTTACCGGCAACTTTC

SUPPLEMENTARY REFERENCES

1 Cox, J. S., Shamu, C. E. & Walter, P. Transcriptional Induction of Genes Encoding Endoplasmic-Reticulum Resident Proteins Requires a Transmembrane Protein-Kinase. *Cell* **73**, 1197-1206, doi:Doi 10.1016/0092-8674(93)90648-A (1993).

2 Thibault, G. *et al.* The membrane stress response buffers lethal effects of lipid disequilibrium by reprogramming the protein homeostasis network. *Mol Cell* **48**, 16-27, doi:10.1016/j.molcel.2012.08.016 (2012).

3 Habeck, G., Ebner, F. A., Shimada-Kreft, H. & Kreft, S. G. The yeast ERAD-C ubiquitin ligase Doa10 recognizes an intramembrane degron. *The Journal of cell biology* **209**, 621, doi:10.1083/jcb.20140808804292015c (2015).

4 Cox, J. S. & Walter, P. A novel mechanism for regulating activity of a transcription factor that controls the unfolded protein response. *Cell* **87**, 391-404 (1996).

5 Prinz, W. A. *et al.* Mutants affecting the structure of the cortical endoplasmic reticulum in Saccharomyces cerevisiae. *The Journal of cell biology* **150**, 461-474 (2000).

6 Merksamer, P. I., Trusina, A. & Papa, F. R. Real-time redox measurements during endoplasmic reticulum stress reveal interlinked protein folding functions. *Cell* **135**, 933-947, doi:10.1016/j.cell.2008.10.011 (2008).

7 Habeck, G., Ebner, F. A., Shimada-Kreft, H. & Kreft, S. G. The yeast ERAD-C ubiquitin ligase Doa10 recognizes an intramembrane degron. *The Journal of cell biology* **209**, 261-273, doi:10.1083/jcb.201408088 (2015).

8 Sikorski, R. S. & Hieter, P. A system of shuttle vectors and yeast host strains designed for efficient manipulation of DNA in Saccharomyces cerevisiae. *Genetics* **122**, 19-27 (1989).

9 Kimata, Y., Oikawa, D., Shimizu, Y., Ishiwata-Kimata, Y. & Kohno, K. A role for BiP as an adjustor for the endoplasmic reticulum stress-sensing protein Ire1. *The Journal of cell biology* **167**, 445-456, doi:10.1083/jcb.200405153 (2004).
10 Snider, J. *et al.* Detecting interactions with membrane proteins using a membrane two-hybrid assay in yeast. *Nat Protoc* **5**, 1281-1293, doi:10.1038/nprot.2010.83 (2010).

UNCROPPED IMMUNOBLOT IMAGES OF MAIN AND SUPPLEMENTARY FIGURES

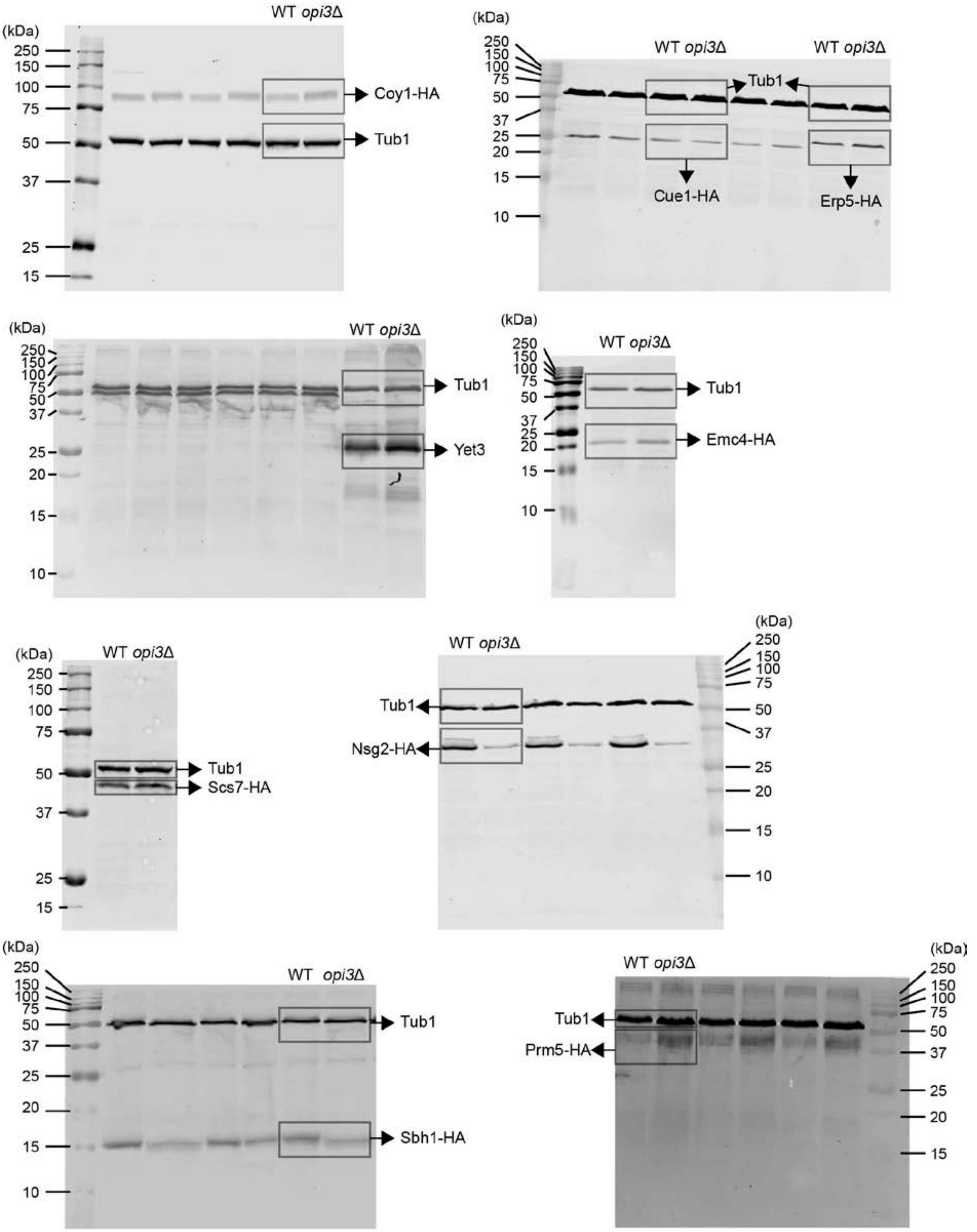

Uncropped images for Figure 1B

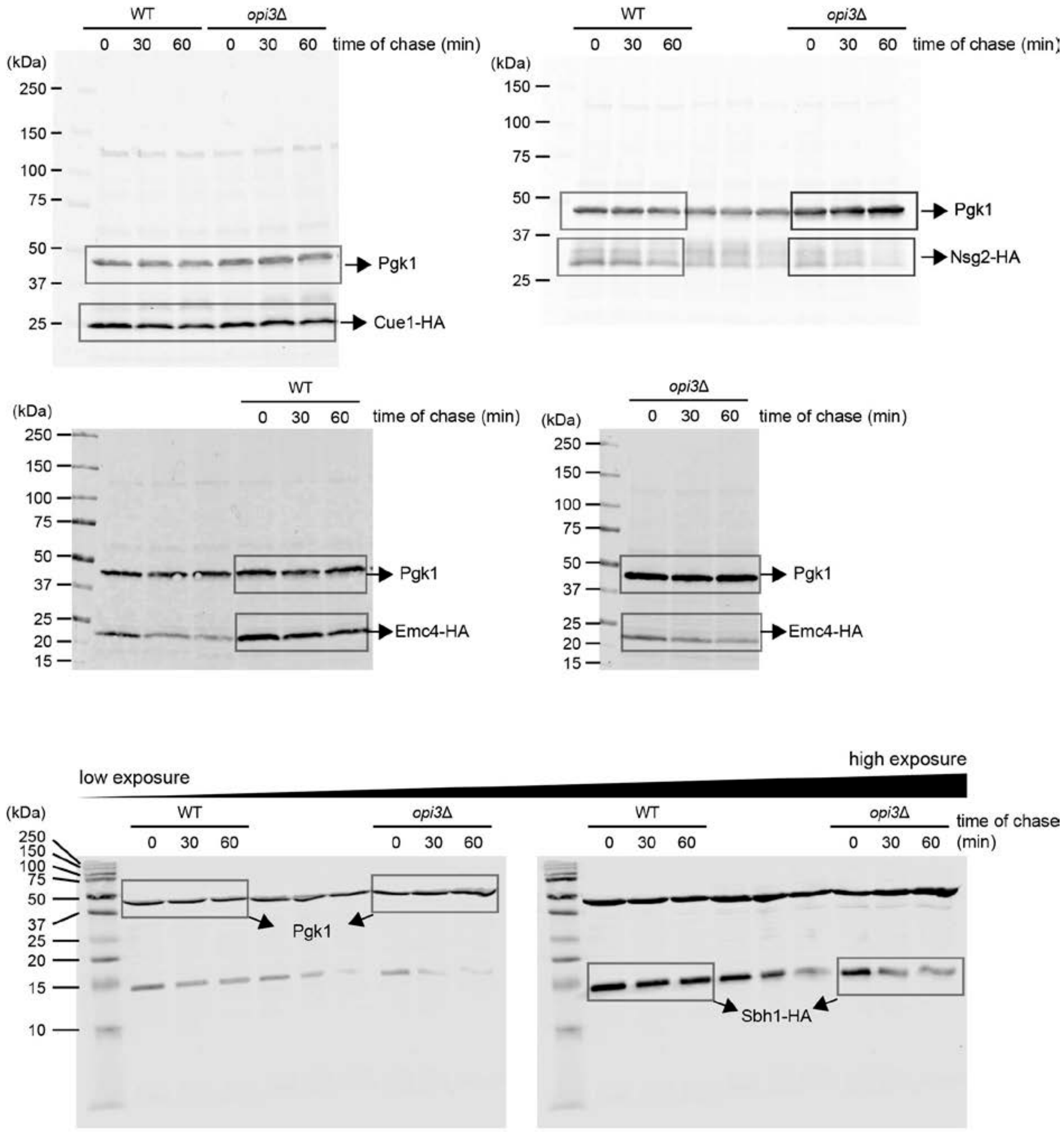

Uncropped images for Figure 1C

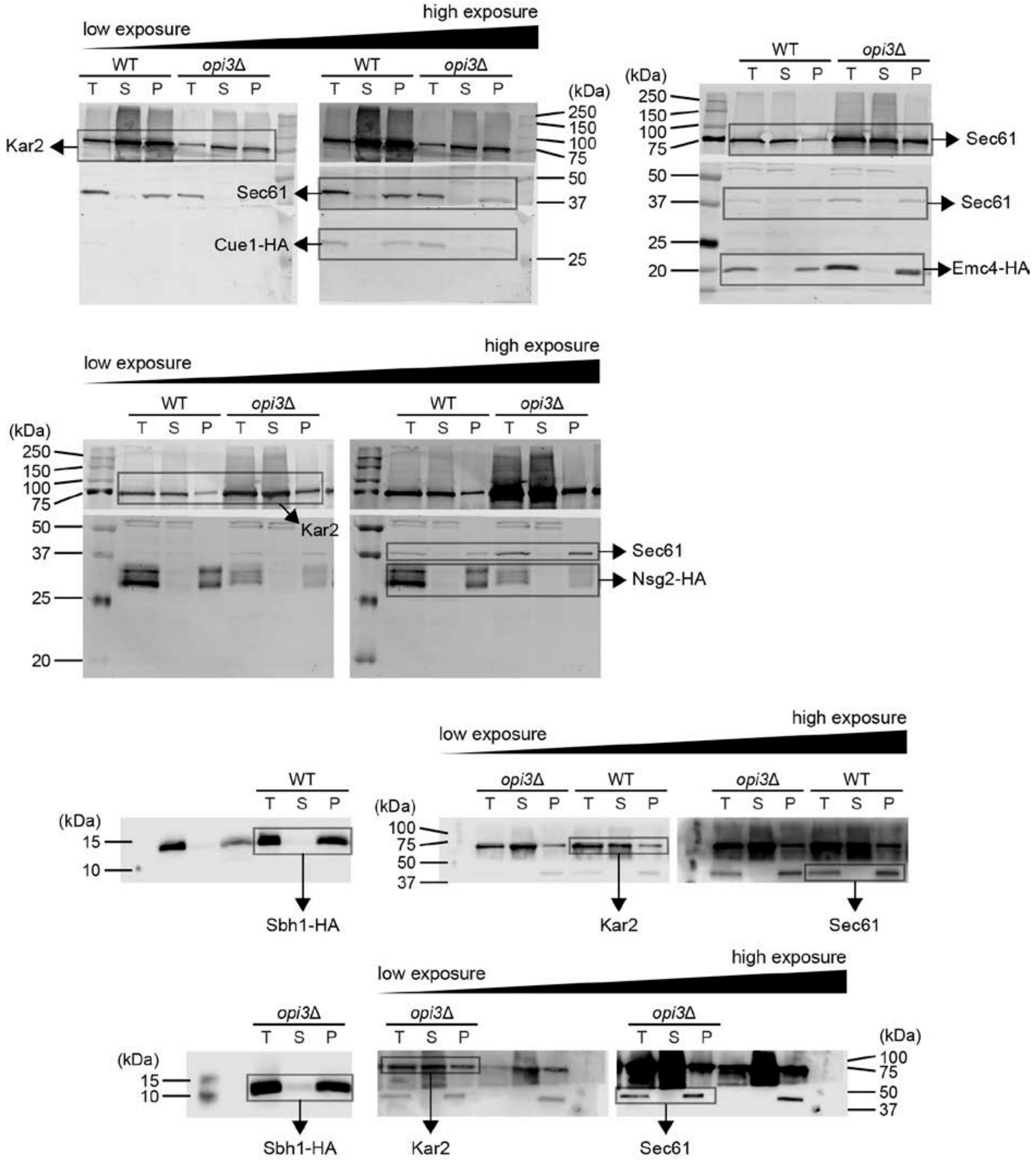

Uncropped images for Figure 2B

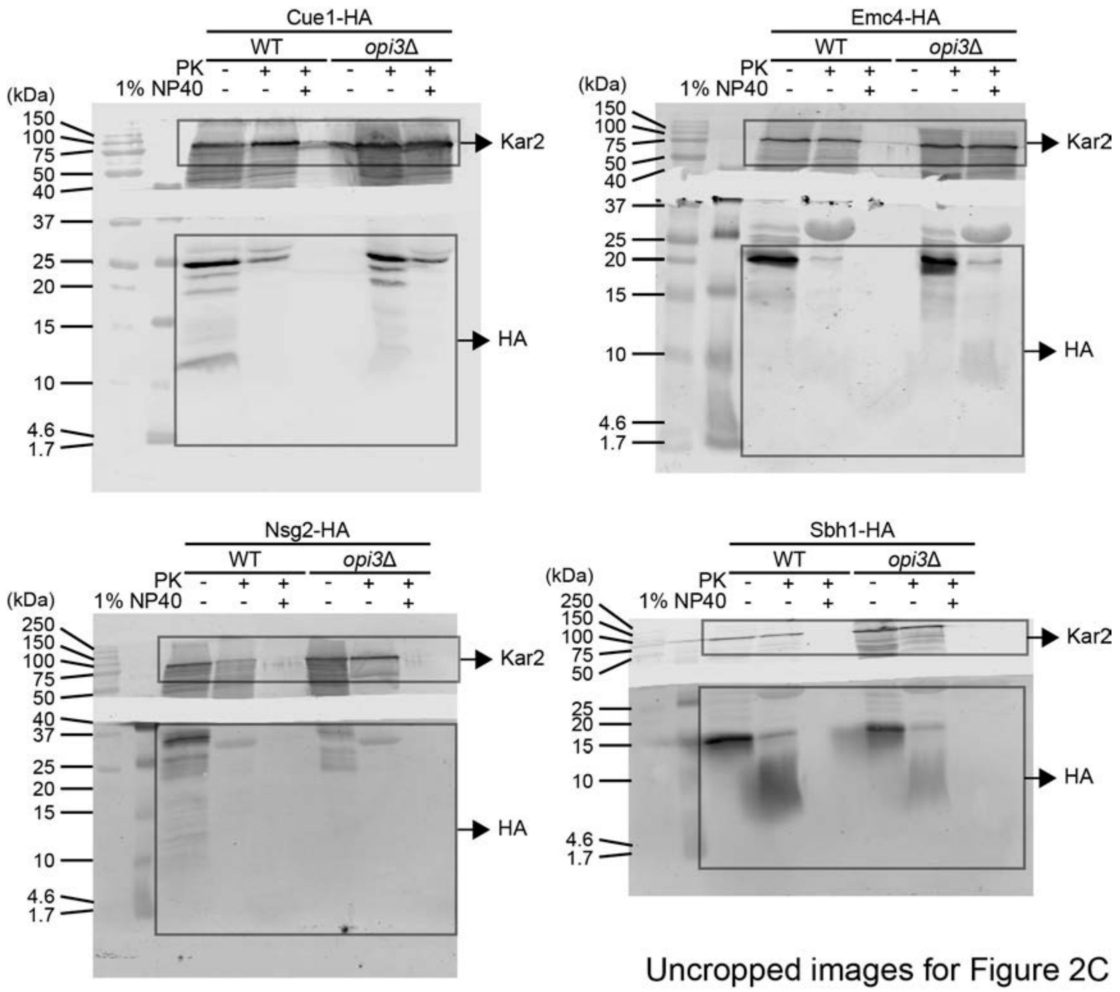

Uncropped images for Figure 2C

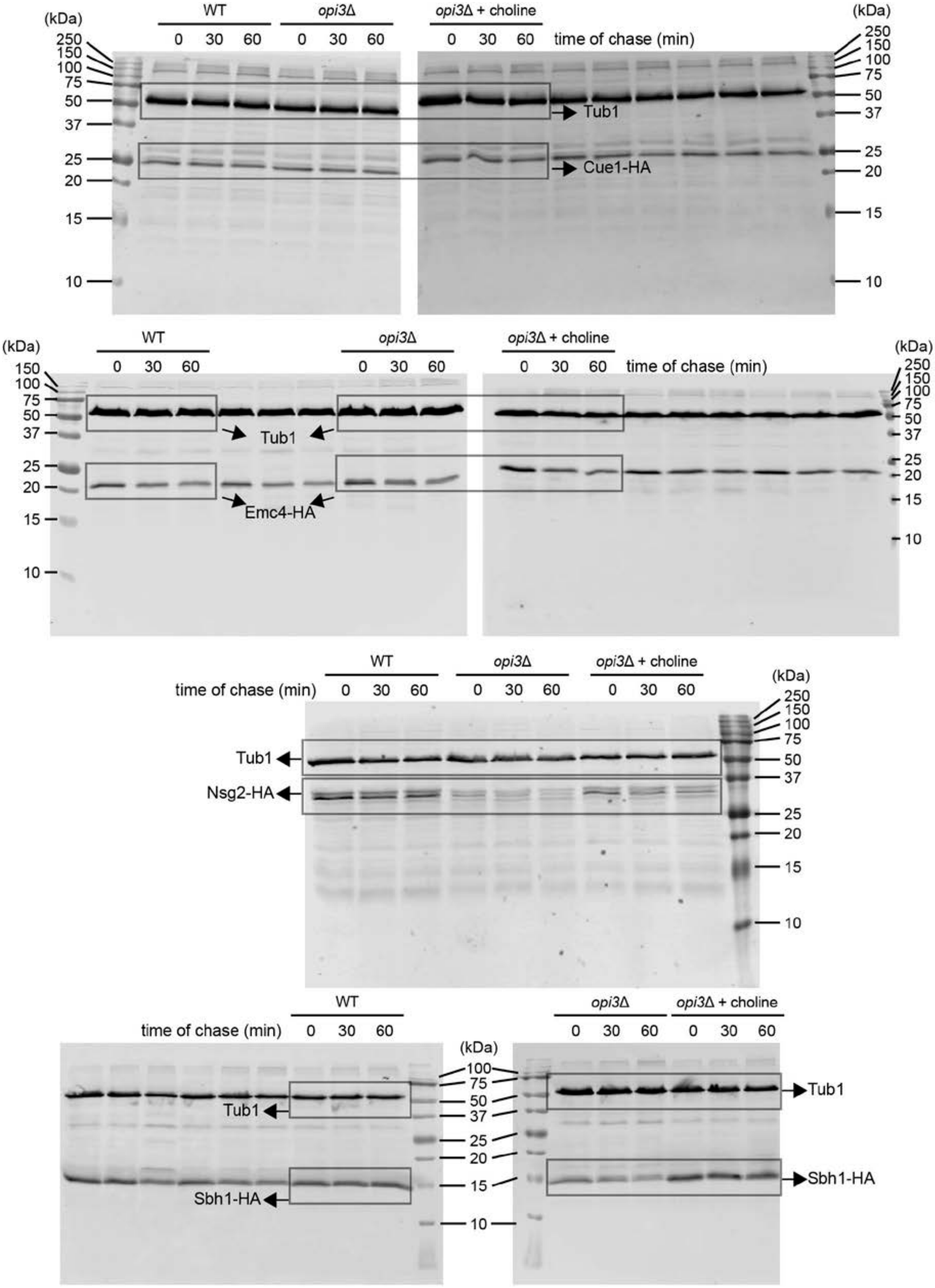

Uncropped images for Figure 3A

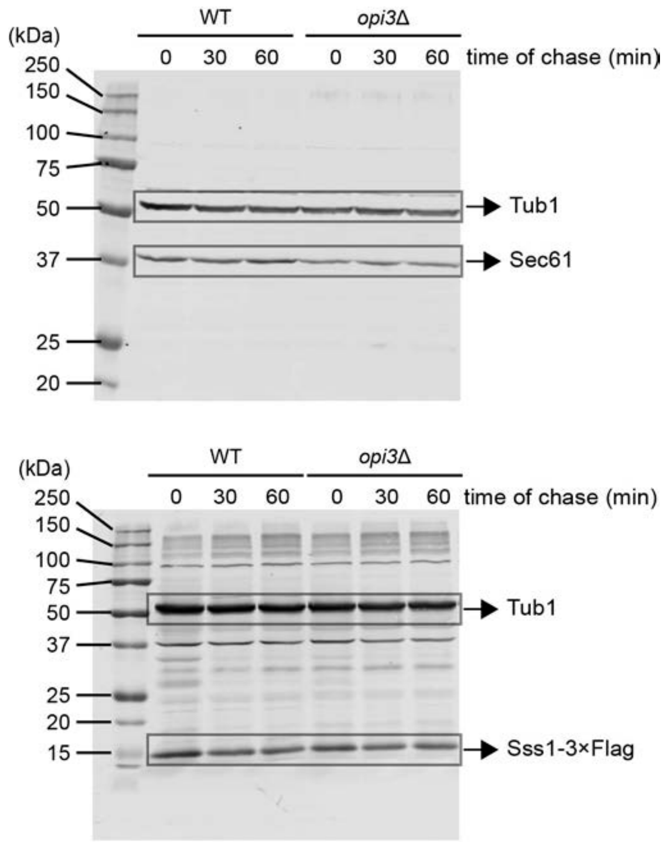

Uncropped images for Figure 4E

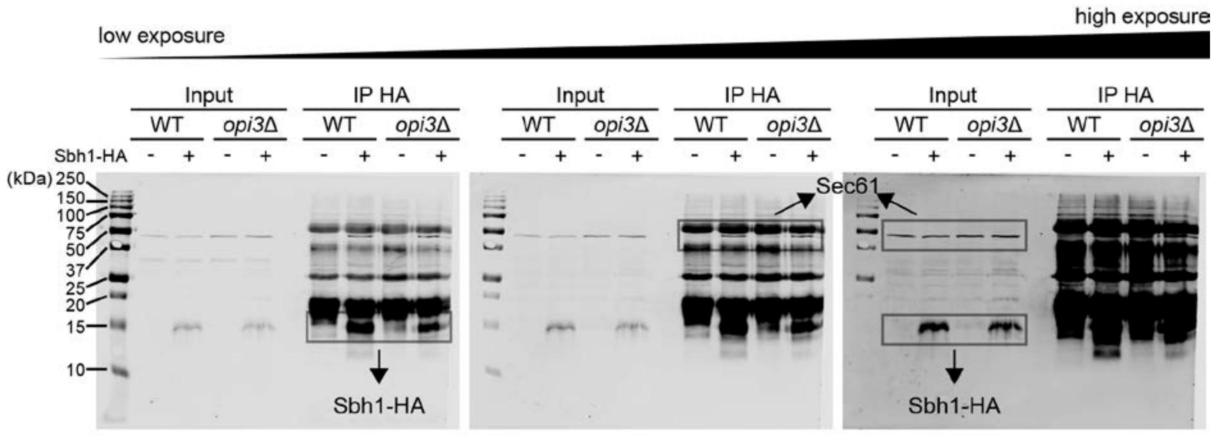

Uncropped images for Figure 4F

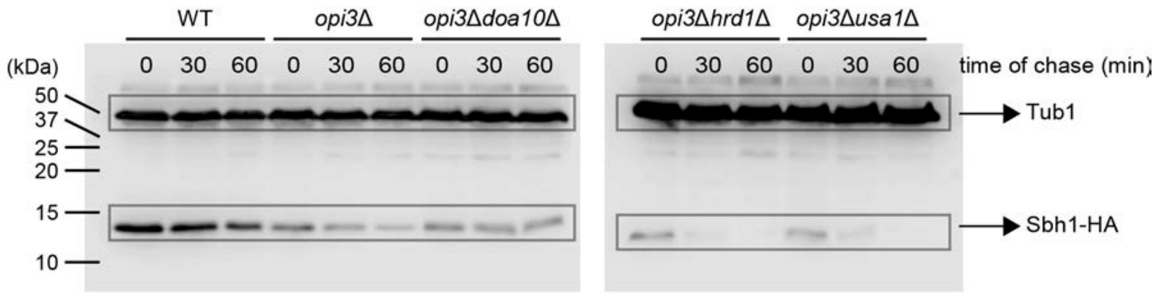

Uncropped images for Figure 5A

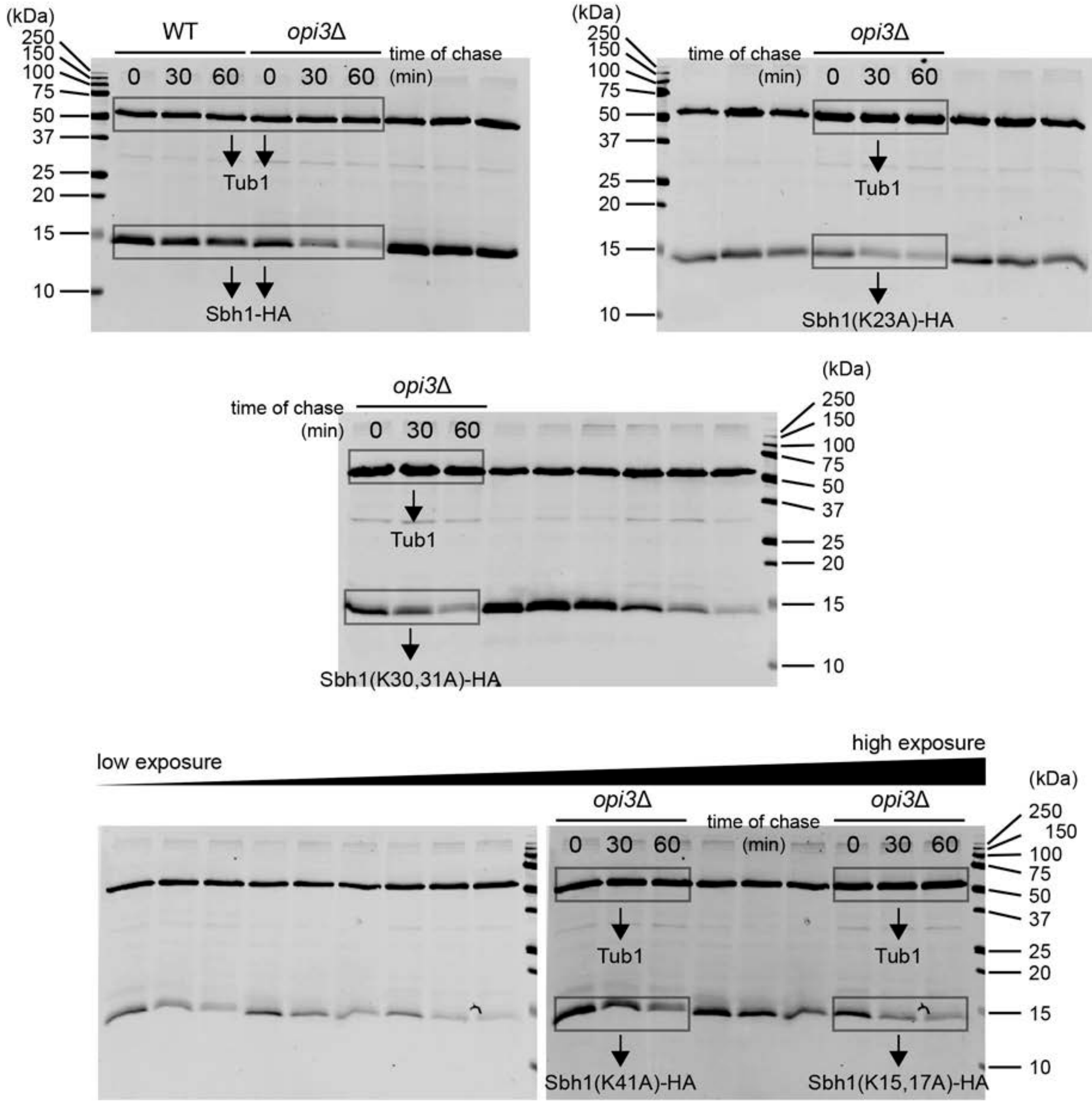

Uncropped images for Figure 5B

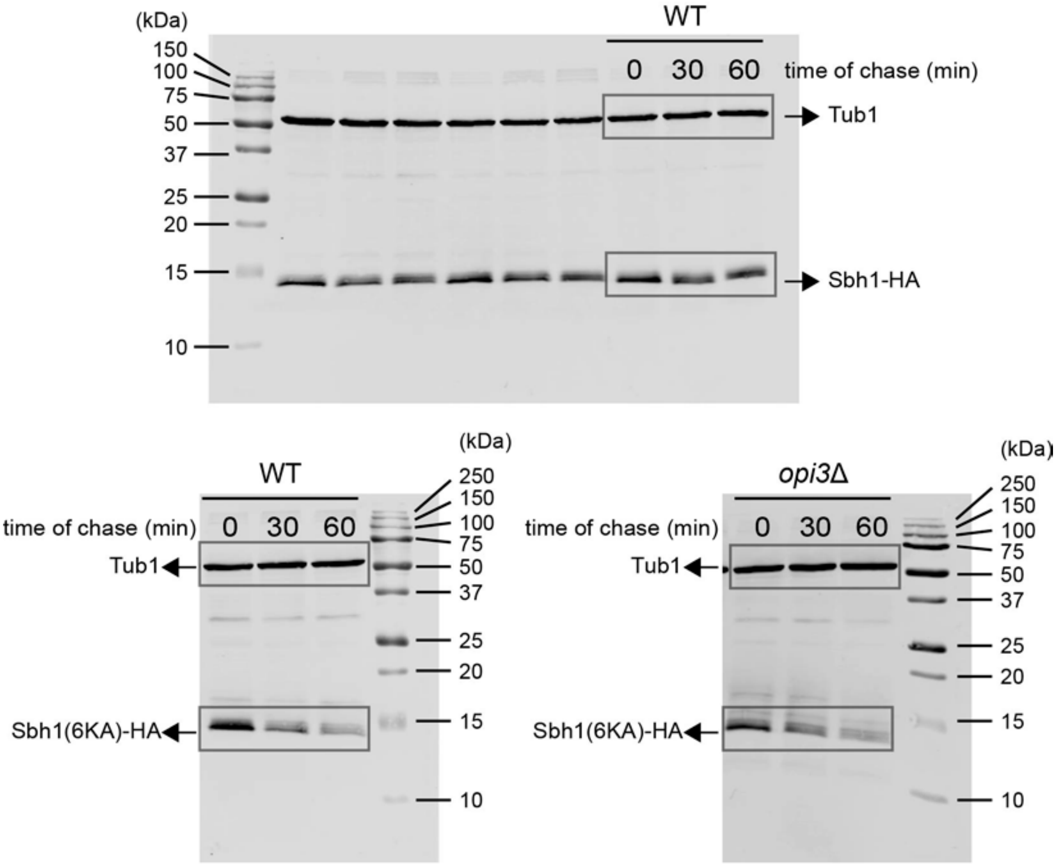

Uncropped images for Figure 5D

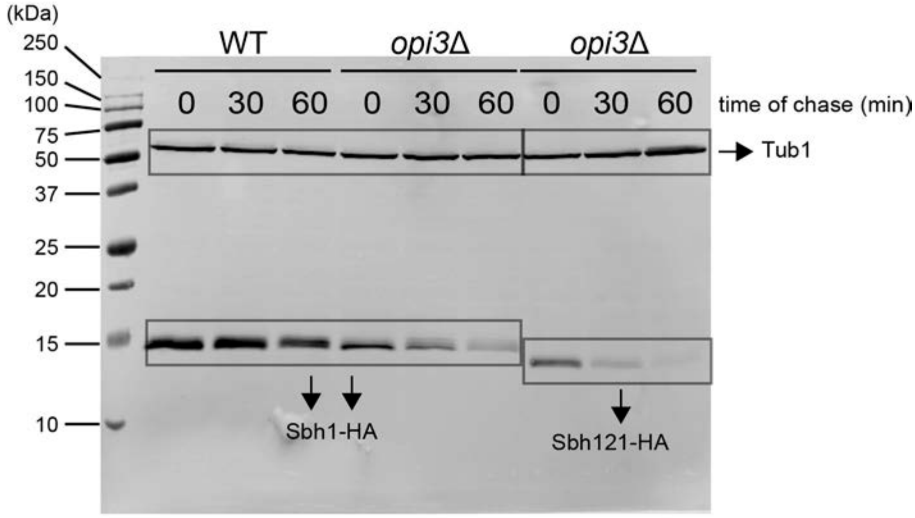

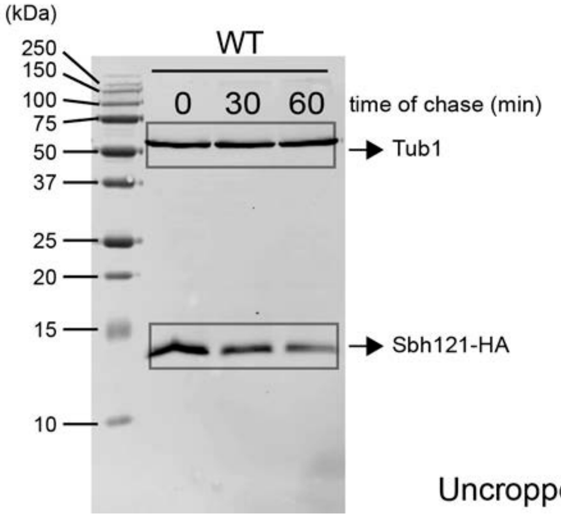

Uncropped images for Figure 5G

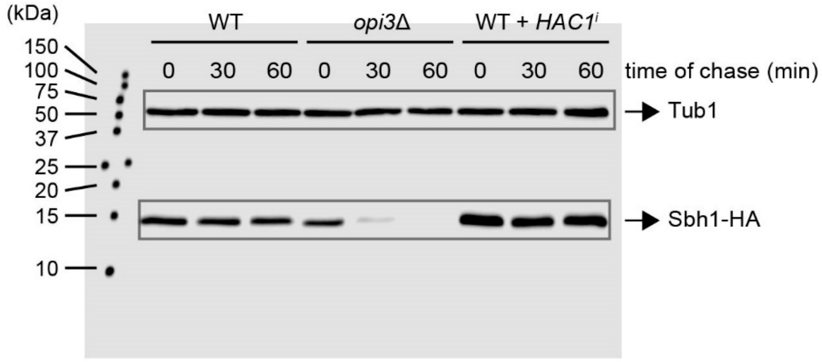

Uncropped images for Figure S1A

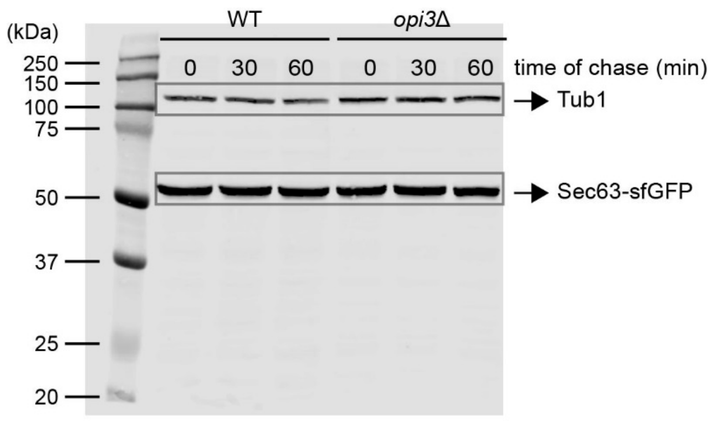

Uncropped images for Figure S2B

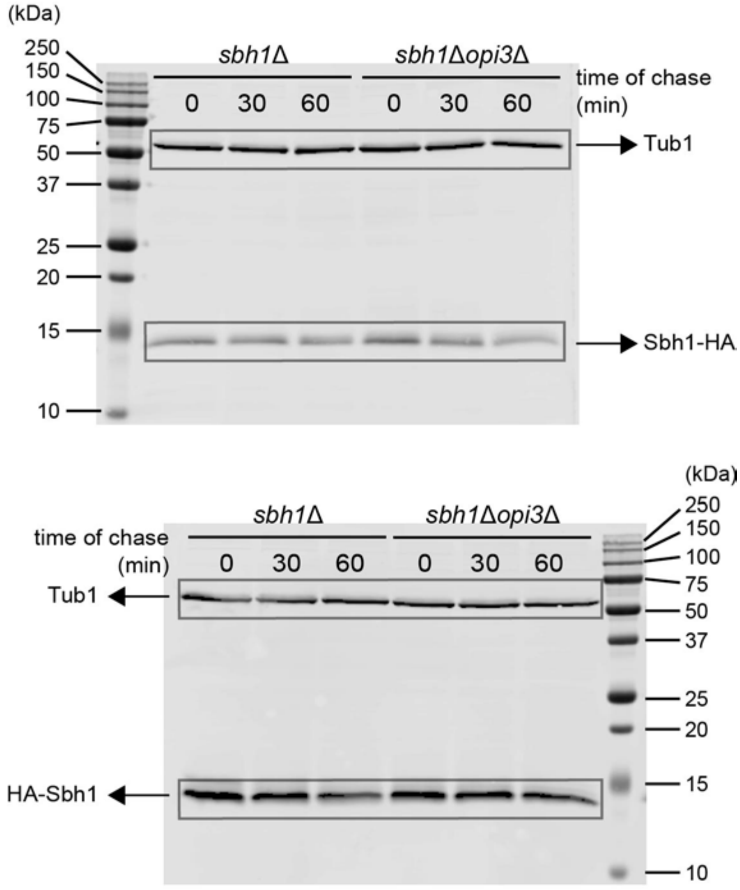

Uncropped images for Figure S3D

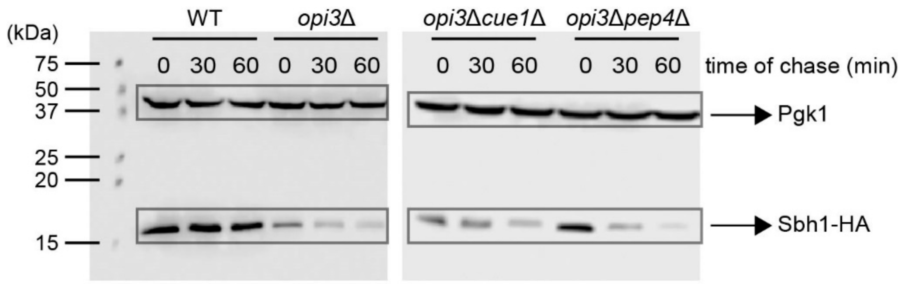

Uncropped images for Figure S4A

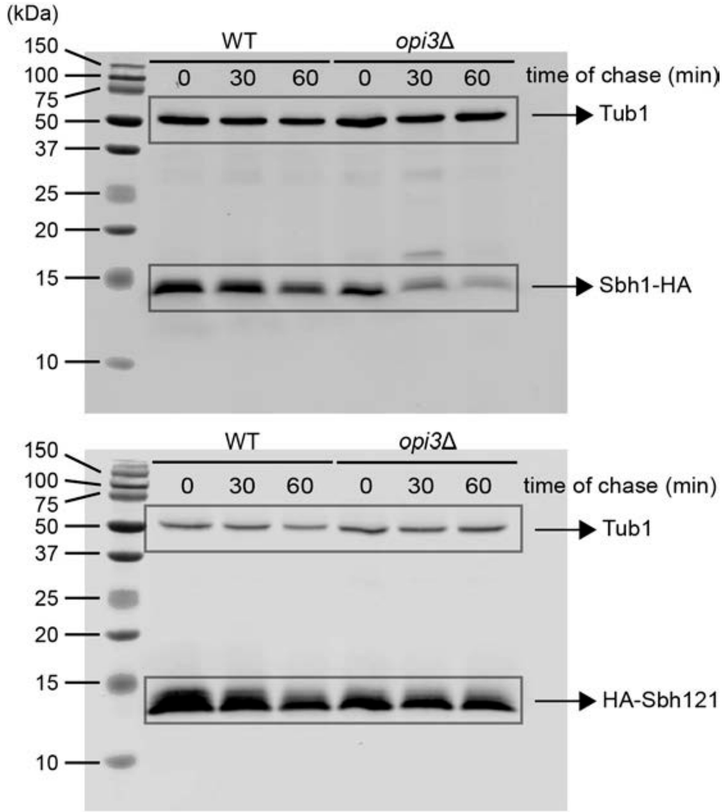

Uncropped images for Figure S4C
